# Exploration for novel inhibitors showing back-to-front approach against VEGFR-2 kinase domain (4AG8) employing molecular docking mechanism and molecular dynamics simulations

**DOI:** 10.1186/s12885-018-4050-1

**Published:** 2018-03-07

**Authors:** Shailima Rampogu, Ayoung Baek, Amir Zeb, Keun Woo Lee

**Affiliations:** Division of Applied Life Science (BK21 Plus Program), Systems and Synthetic Agrobiotech Center (SSAC), Plant Molecular Biology and Biotechnology Research Center (PMBBRC), Research Institute of Natural Science (RINS), Gyeongsang National University (GNU), 501 Jinju-daero, Jinju, 52828 Republic of Korea

**Keywords:** Angiogenesis, Progression, Back-to-front approach, VEGFR-2, MD simulations

## Abstract

**Background:**

Angiogenesis is a process of formation of new blood vessels and is an important criteria demonstrated by cancer cells. Over a period of time, these cancer cells infect the other parts of the healthy body by a process called progression. The objective of the present article is to identify a drug molecule that inhibits angiogenesis and progression.

**Methods:**

In this pursuit, ligand based pharmacophore virtual screening was employed, generating a pharmacophore model, Hypo1 consisting of four features. Furthermore, this Hypo1 was validated recruiting, Fischer’s randomization, test set method and decoy set method. Later, Hypo1 was allowed to screen databases such as Maybridge, Chembridge, Asinex and NCI and were further filtered by ADMET filters and Lipinski’s Rule of Five. A total of 699 molecules that passed the above criteria, were challenged against 4AG8, an angiogenic drug target employing GOLD v5.2.2.

**Results:**

The results rendered by molecular docking, DFT and the MD simulations showed only one molecule (Hit) obeyed the back-to-front approach. This molecule displayed a dock score of 89.77, involving the amino acids, Glu885 and Cys919, Asp1046, respectively and additionally formed several important hydrophobic interactions. Furthermore, the identified lead molecule showed interactions with key residues when challenged with CDK2 protein, 1URW.

**Conclusion:**

The lead candidate showed several interactions with the crucial residues of both the targets. Furthermore, we speculate that the residues Cys919 and Leu83 are important in the development of dual inhibitor. Therefore, the identified lead molecule can act as a potential inhibitor for angiogenesis and progression.

**Electronic supplementary material:**

The online version of this article (10.1186/s12885-018-4050-1) contains supplementary material, which is available to authorized users.

## Background

Initiation of tumour cell and its progression is a process which is performed by certain factors known as angiogenic factors [1]. Angiogenesis is a complex process during which the endothelial cells are involved in the generation of metallo-proteases, initiate cell migration, cell division, and further proliferation. Additionally, they are also responsible for the formation of the new cells [2]. More specifically, cancer has an ability of rapid cell growth and hence, it is evident that angiogenesis supports the cancer metastasis [1].It is therefore essential to identify the novel drug molecules, which could hinder angiogenesis.

Vascular Endothelial Growth Factors (VEGFs) demonstrate an essential role in angiogenesis and vasculogenesis [3] and therefore, portray to be an ideal drug targets for designing novel inhibitors. Typically, VEGFRs are the transmembrane proteins that are known to trigger angiogenesis through VEGF receptor signalling [4]. There are three different types of receptor tyrosine kinases (RTK) which display a high affinity towards VEGF, namely, VEGFR-1, VEGFR-2 and VEGFR-3, respectively. However, VEGFR-2 remains as the only protein kinase domain transmitting the angiogenic signals [5], while the VEGFR-1 revealed a reduced activity than VEGFR-2 [6, 7] and VEGFR-3 exhibits its role in governing the embryonic angiogenesis [8]. Therefore, VEGFR-2 emerges as an ideal protein target to identify new drugs. Moreover, VEGFR-2 has a crucial role in rheumatoid arthritis [9] inflammation [10] porosis [11], metastasis [12] and ocular neovascularization [13]. Accordingly, identification of novel drugs against VEGFR-2 might also have a curative effect on the aforementioned diseases.

Depending upon the binding patterns, the tyrosine kinase inhibitors can be grouped into type I and type II [14]. Type I inhibitors interact with the Adenosine Triphosphate (ATP) binding site of the kinase in its active form [15] and thereby, displaying reduced selectivity, while in type II, the inhibitors bind to the ATP site along with the allosteric, hydrophobic site [16] and exhibits high selectivity. This interaction happens during the inactive state of the kinases [17]. The conserved triad Asp-Phe-Gly (DGF) governs the active and the inactive states of the kinase enzymes. Generally, DGF-in conformation was noticed in the active condition, whereas in the DFG-out is projected during the inactive state [17]. The simple architecture of VEGFR-2 active site comprises of the front and the back pocket. ATP- binding front pocket has two key residues associated with it, Glu917 and Cys919. The back hydrophobic pocket has Glu885 and Asp1046. Glu885 is seated on the αC helix and the Asp1046 forms an important part of the triad [18, 19].

One of the highly significant characters of the cancer cells is being able to divide rapidly [20]. Cyclic-dependent kinases (CDKs), are crucial enzymes and contribute at large towards this process and belong to the serine-threonine kinases subfamily [21]. These kinases have gained popularity for their role in cell division, differentiation, neuronal functions, transcription and apoptosis [22]. Of all the known CDKs, CDK1-CDK13, CDK2 is widely studied protein as it has a crucial role to be played during the cell cycle progression more specifically during the G1 to S phase transition [22, 23]. However, this requires being associated with regulatory subunits such as cyclin A and E [23]. Cyclin A is complexed with CDK2 for the S-phase (Synthesis phase) progression while cyclin E is required during the retinoblastoma protein phosphorylation that provides the transition of G1/S (Gap1) [23, 24]. Additionally, reports exists on the inhibition of CDK2 that can eventually lead to the killing of the cancerous cells by deregulation of E2F-1 activity [25]. Therefore, it will be beneficial to identify a small molecule that can act both on the angiogenesis and on progression. Such a type of inhibition was earlier reported by Antony et al. [26].

The objective of the present study is to identify a novel inhibitor that has a potential to interact both with the VEGFR protein and with the CDK2 protein. In the present investigation, an attempt was made to screen inhibitors that bind more precisely in the back-to-front fashion of the target protein. This approach has been proven extremely successful with respect to certain kinases [27–29].

Although, both the proteins share a very little homology, they exhibit structural similarity within the ATP- binding region. Accordingly, the current study tries to exploit these important features in identifying the novel dual drug/inhibitor. The investigation proceeds with the initial identification of VEGFR-2 inhibitors and then challenging them with the CDK2 protein.

## Methods

### Preparation of the dataset

**Fig. 1:**
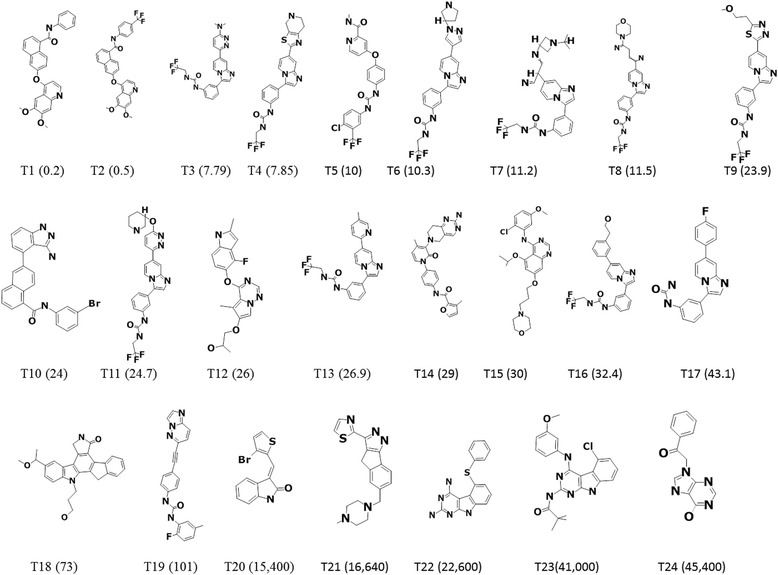
2D structures of 24 training set compounds and their IC_50_ values represented in parenthesis

A systematic literature survey was conducted to extract the dataset for VEGFR-2 inhibitors [30]. From the obtained information of 63 diverse compounds [31–39], 24 structurally diverse compounds were referred to as training set and the remaining 39 were grouped into test set compounds. The training set compounds were employed to build the pharmacophore model while the test set was used to validate the same. The important criteria in choosing the training set compounds is the inclusion of the most active compounds into it such that they impart the most reliable information pertaining to the generated pharmacophore model. Additionally, the training set compounds exhibited a wide range of, half maximal inhibitory concentration, IC_50_, spanning between 0.2 to 45,000 nmol/L. Further, the compounds in the training set, (Fig. 1), were designated as most active (IC_50_ < 250 nmol/L, +++), moderately active (250 nmol/L ≤ IC_50_ < 5000 nmol/L, ++) and inactive (IC_50_ ≥ 5000 nmol/L, +), that was adapted based on the IC_50_values. The same criteria were followed for the test set compounds. Their corresponding two dimensional (2D) structures were sketched using the ChemSketch (http://www.acdlabs.com/ resources/ freeware/ chemsketch/ Advanced Chemistry Development (ACD) Inc., Toronto, Canada) and were translated to their three dimensional (3D) structures adapting the Discovery Studio v4.5 (DS).

### Generation of the pharmacophore model

In the recent times, pharmacophore modelling takes advantage of being one of the most reliable methods in identifying novel leads for different targets. For the present investigation, the three dimensional quantitative structure-activity relationship (3D–QSAR) based pharmacophore model was generated using the Catalyst *HypoGen* algorithm provided with the DS v4.5. This exploits the chemical features of the training set compounds and the conformation with the least energy were developed employing the *BEST* algorithm. In order to generate the best pharmacophore model, the energy and the uncertainty value were fixed at 20 kcal/mol and 3, respectively [40]. Further, *Feature Mapping* protocol was employed for investigating into the chemical features and to recognize the common features present in the training set that could be essential in the pharmacophore generation. This protocol has an ability to construct pharmacophore features available with the training set compounds and further these identified features play a critical role in the generation of the model. Amongst the generated models, the best hypothesis was chosen based upon the Debnath’s method [41].

### Validation of the generated pharmacophore model

With an aim to determine the predictive ability and its capability to identify the active compounds from that of the inactives, the selected pharmacophore was subjected to validation recruiting three different approaches such as, Fischer’s randomization, test set method, and the decoy set method. Fischer’s randomization was carried out alongside the pharmacophore generation, which prompts random spreadsheets based upon the selected level of confidence. For the present investigation, the confidence level was chosen to be 95%. The test and the decoy method of validations were conducted in order to understand if the generated pharmacophore was able to select the compounds in a similar manner as for the experimental activities. *Ligand Pharmacophore Mapping* protocol available on the DS was employed with *Best Flexible* algorithm. Test set was assembled with 39 structurally different compounds. The decoy set was instituted with a database of 710 compounds consisting of 15 active compounds. Following this, the enrichment factor (EF) and the goodness of fit score (GF) were computed using the formulae,

$$ GF=\left[\left(\frac{Ha}{4 HtA}\right)\left(3A+ Ht\right)\ \left(1-\frac{Ht- Ha}{D-A}\right)\right] $$

$$ EF=\frac{\left( Ha\ X\ D\right)}{\left( Ht\ X\ A\right)} $$

Here, Ha represents the total number of active compounds, Ht refers to total hits redeemed from the database, A refers to the total number of active compounds in the database, and D denotes the total number of molecules in the database.

### Database screening for extracting the candidate compounds

Validated pharmacophore was employed as the 3D query to screen the databases such as Maybridge, Asinex, Chembridge, and NCI to retrieve novel scaffolds against angiogenesis, if the chemical compounds mapped with all the chemical features present in the pharmacophore. In this pursuit, the *Ligand Pharmacophore Mapping* protocol was used with *Best–Flexible* options.

### Drug-likeness assessment

Drug-likeness assessment was performed to the retrieved compounds from the databases so as to assess their biological activities. Accordingly, to judge the compound for strong pharmacokinetic properties, ADMET [42] and Lipinski’s rule were applied. ADMET specifically evaluates if the compound can cross the Blood Brain Barrier (BBB), allowable solubility, good intestinal absorption with less toxicity. Therefore, the values 3, 3 and 0 were fixed for BBB, solubility and absorption, correspondingly and were computed adapting *ADMET Descriptors* module on the DS. Additionally, the Lipinski’s Rule of 5 [43] was applied to the above filtered compounds to quantify if the prospective drug molecules could be well absorbed. This rule recommends a compound should have less than 10 hydrogen bond acceptors, less than 5 hydrogen bond donor groups having a molecular weight of less than 500 Da with log *p* value of less than 5 with 10 rotatable bonds. All the compounds that satisfied the aforementioned criteria were forwarded for the docking studies.

### Molecular docking studies

Challenging the potential screened lead molecules with the reliable drug target and to assess the degree of their binding affinities rendered in terms of the dock scores happens to be one of the most significant methodologies in drug discovery. Typically, this approach was deduced to assess the nature of the lead molecules in the active site and thereby its conformation. For the current study, Genetic Optimization for Ligand Docking v5.2.2 (GOLD) has been recruited [44, 45]. Target protein with the protein data bank (PDB) code, 4AG8, with high resolution of 1.95 Å, co-crystalled with axitinib was selected. Missing residues of the protein were rectified and all the hydrogen atoms were added, removing the water molecules [46]. Histidine tautomer orientations were placed in agreement with the crystal structure. The binding site of the protein was calculated for all the atoms that lie within the bound ligand around 15 Å. Furthermore, GOLD score was specified to understand the binding affinities between the ligand and the drug target, while the Chemscore was adapted for enumerating the rescoring function. Moreover, the GOLD score was initiated to generate 50 docking poses for each ligand and the reliable pose was selected based upon the highest dock score, molecular interactions and the hydrogen bonds that resulted between the ligand and the amino acid residues present at the active site of the protein molecule. Hereinafter, the most active compound in the training set was labeled as the reference compound.

The lead molecules identified after challenging against the 4AG8 would be further challenged with the CDK2 protein, 1URW, a potential target for cancer progression.

### Density functional theory

DFT is one of the most dynamic methods adapted to calculate the electronic structure of matter and thus provides the most valuable information with respect to the selected inhibitors. DFT for the resultant docking molecules was performed using Becke, 3-parameter, Lee-Yang Parr (B3LYP) [47], available on the DS in order to evaluate their orbital energies such as highest occupied molecular orbitals (HOMO) and lowest unoccupied molecular orbitals (LUMO). HOMO refers to the electron donor and LUMO denotes the electron acceptor. DFT was executed together with the Hit compounds and the compounds from the training set.

### Molecular dynamics simulations

To gain further insight into the protein-ligand interactions, the procured Hit compounds from the docking studies and the DFT were subjected to the Molecular Dynamics (MD) simulations along with the reference compound. The ligand topologies were generated utilizing the SwissParam [48–50], while the topologies of the protein were generated employing Chemistry at HARvard macromolecular Mechanics force-field (CHARMm ff) [51–54] implemented in Groningen Machine for Chemical Simulations (GROMACS) 5.0.6 [55]. Dodecahedron box was obtained and was solvated with three-site transferable intermolecular potential (TIP3P) water model followed by neutralizing the system with the counter ions. All the bad contacts were further removed by subjecting the system to pass through steepest descent algorithm at 10,000 steps with an upper limit of the force being lower than 1000 kJ/mol [56]. Following this, the equilibration was conducted by Number of particles, Volume and Temperature (NVT) [57] and Number of particles, Pressure and Temperature (NPT) [58] at 100 ps at 300 k and 100 ps at a pressure of 1 bar maintained by Parrinello-Rahman barostat and allowing the movement of the counter ions and the water molecules, constraining the protein backbone. Linear Constraint Solver for Molecular Simulations (LINCS) [59] algorithm was used to restrain heavy atom bonds and their respective hydrogen atoms. Particle Mesh Ewald (PME) [60] was utilized to compute the long rage electrostatic interaction and a cut-off distance of 12 Å was attributed for Coulombic and van der Waals interactions. MD simulations were performed for 30 ns storing the coordinate data for every 2 fs. Corresponding results were evaluated employing the Visula Molecular Dynamics (VMD) [61] and DS, respectively.

## Results

### *HypoGen* based pharmacophore model generation

**Fig. 2:**
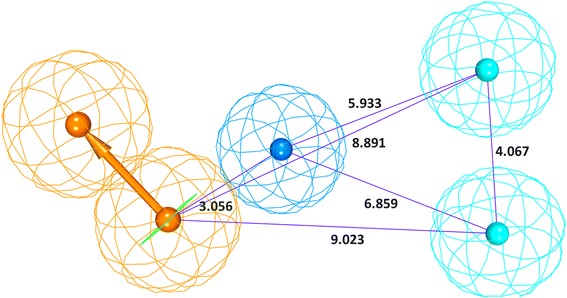
Hypo1 pharmacophore model with its corresponding features and geometry, Aromatic Rings (brown), Hydrophobic Aliphatic (blue), 2 Hydrophobic (cyan)

Using the *HypoGen* algorithm provided with the DS, 24 training set compounds were employed to develop the pharmacophore model, (Figs. 1 and 2), which resulted in the generation of 10 hypotheses, Table 1, upon the utilization of *3D QSAR Pharmacophore Generation* protocol available with the DS. The preferred features for the pharmacophore generation were hydrogen bond acceptor (HBA), hydrogen bond donor (HBD), hydrophobic (HyP), hydrophobic aliphatic (Hy-Ali) and ring aromatic (RA).

**Table 1:** Statistical data of the generated hypothesis employing HypoGen

Hypo no	Total cost^a^	Cost difference	RMSD^b^	Correlation	Features^c^	Max fit
Hypo1	111.95	71.22	0.7	0.97	HyAli, 2HyB,RA	11.4
Hypo 2	113.31	69.86	0.7	0.96	HyAli, 2HyB,RA	11.5
Hypo 3	116.45	66.71	0.8	0.95	HyAli,HyB,RA,HBA	11.9
Hypo 4	116.47	66.69	1.0	0.94	HBA, HBD 2HyB	10.7
Hypo 5	117.11	66.05	0.9	0.94	HyAli,HyB,RA,HBA	11.5
Hypo 6	119.51	63.65	1.0	0.93	HBA,HBD,2HyB	11.26
Hypo 7	119.52	63.65	0.9	0.95	HBA,2HyB,RA	12.65
Hypo 8	119.82	63.35	0.9	0.94	HBA,Hy-Ali,HD,RA	12.33
Hypo 9	119.94	63.23	1.2	0.91	HBA,Hy-Ali,2HyB, RA	11.98
Hypo10	120.52	62.65	1.1	0.91	HBA,HBD,HY-Ali	7.8

^a^Cost difference between the null and the total cost. The null cost, the fixed cost and the configuration cost were found to be 183.177, 101.77 and 19.91, respectively

^b^RMSD-Root Mean Square Deviation

^c^HyP- Hydrophobic, RA- Ring Aromatic, Hy-Ali-Hydrophobic Aliphatic, HBA-Hydrogen Bond Acceptor, HBD-Hydrogen Bond Donor

From the generated models, Hypo1 was chosen as the best pharmacophore model as it satisfied the Debnath’s rules, which states that a good pharmacophore model should consists of high cost difference, good correlation, least RMSD and low cost values. The generated pharmacophore model composed of aromatic feature (RA), one hydrophobic aliphatic feature (Hy-Ali) and two hydrophobic (HyP) features.

**Fig. 3:**
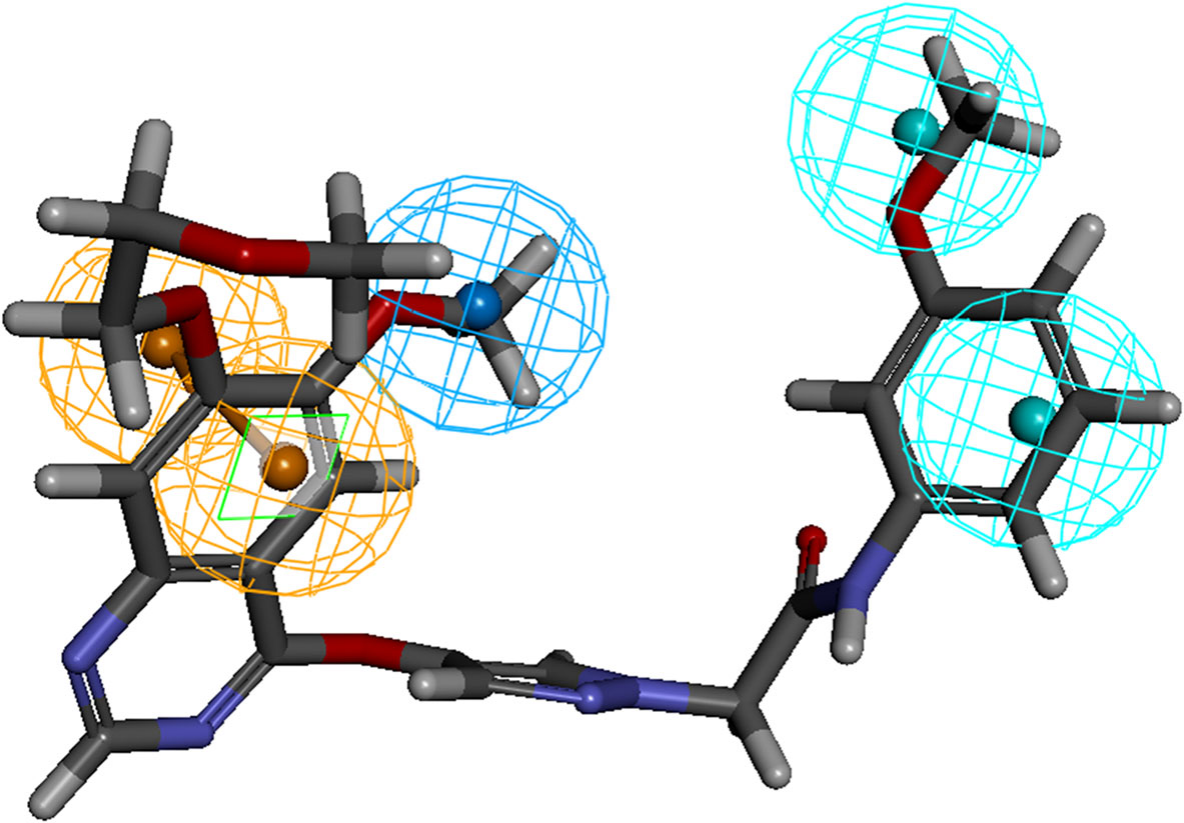
Most active compound (IC_50_ = 0.2) mapped to all the features

**Fig. 4:**
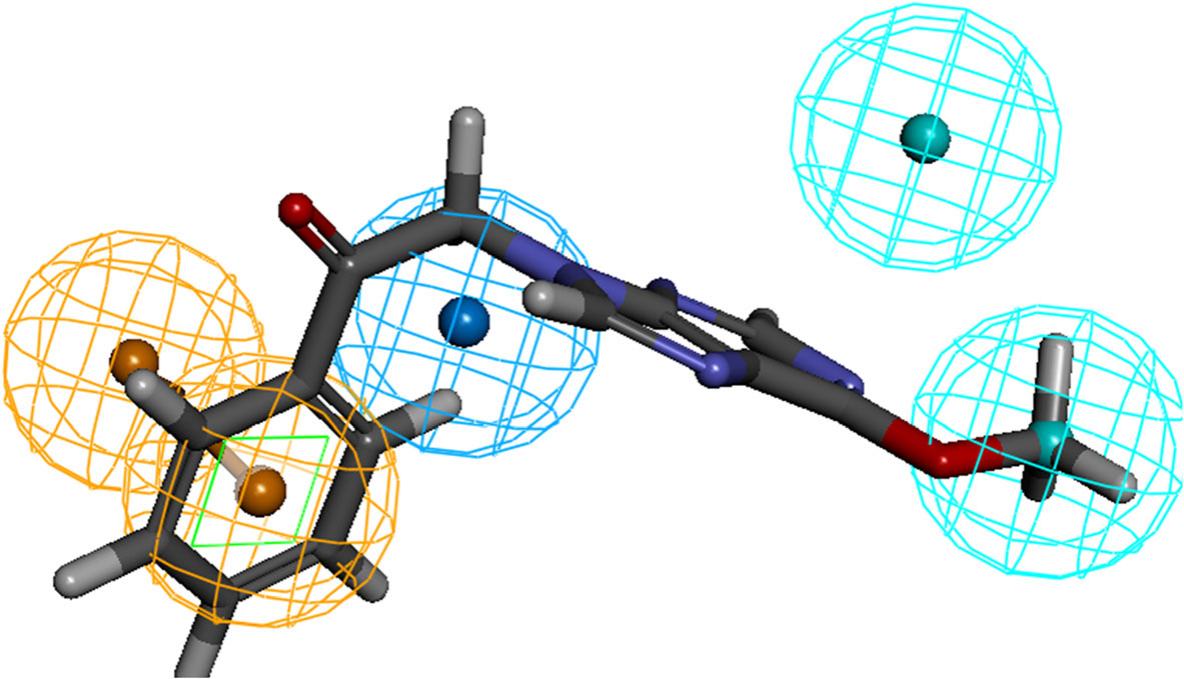
Most inactive compound (IC_50_ = 45,000) aligned to only three features

Further, to evaluate the predictive ability of the Hypo1, it was allowed to examine the inhibitory activities of 24 training set compounds. The training set compounds were grouped into most active, moderately active and the least active compounds based upon their IC_50_ values as, (IC_50_ < 250 nmol/L, +++), (250 nmol/L ≤ IC_50_ < 5000 nmol/L, ++) (IC_50_ ≥ 5000 nmol/L, +), correspondingly. Hypo1 calculated the inhibitory activity values of the training set in accordance with the experimental values, Table 2. Furthermore, Hypo1 has successfully mapped to the most active compound and the most inactive compound, (Figs. 3 and 4).

**Table 2:** Assessing the training set compound values for estimated and the experimental activities by Hypo1

Name	Fit value	Experimental IC50 (nmol/L)	Predicted IC 50 (nmol/L)	Error^a^	Experimental scale	Predicted scale
Molecule1	10.47	0.2	0.191	−1.04	+++	+++
Molecule2	8.58	5.2	14.9	2.86	+++	+++
Molecule3	8.40351	7.79	22.7	2.91	+++	+++
Molecule4	8.4847	7.85	18.83	2.39	+++	+++
Molecule5	7.6927	10	116.68	11.66	+++	+++
Molecule6	8.4082	10.3	22.46	2.18	+++	+++
Molecule7	8.95463	11.2	6.38	−1.75	+++	+++
Molecule8	8.44562	11.5	20.61	1.79	+++	+++
Molecule9	8.42254	23.9	21.73	−1.09	+++	+++
Molecule10	8.004	24	56.97	2.37	+++	+++
Molecule11	8.36497	24.7	24.816	1	+++	+++
Molecule12	8.50932	26	17.79	−1.46	+++	+++
Molecule13	8.20893	26.9	35.54	1.32	+++	+++
Molecule14	7.99567	29	58.08	2	+++	+++
Molecule15	8.48967	29.51	18.62	−1.58	+++	+++
Molecule16	8.36485	32.4	24.82	−1.3	+++	+++
Molecule17	8.19005	43.1	37.12	−1.16	+++	+++
Molecule18	7.79069	73	93.11	1.27	+++	+++
Molecule19	8.47375	101	19.31	−5.22	+++	+++
Molecule20	5.74562	15,400	10,329.50	−1.49	+	+
Molecule21	5.23885	16,600	33,178.10	1.99	+	+
Molecule22	5.71617	22,600	11,054.20	−2.04	+	+
Molecule23	5.7456	41,200	10,330.10	−3.98	+	+
Molecule24	5.74648	45,000	10,309.20	−4.36	+	+

^a^Error, ratio of the predicted activity (Pred IC50) to the experimental activity (Exp IC50) or its negative inverse if the ratio is < 1

### Validation of the pharmacophore model, Hypo1

In order to assess the quality of the generated pharmacophore, it was subjected to a series of validations such as, Fischer’s randomization method, test set method and the decoy test method.

#### Fischer’s randomization method

**Fig. 5:**
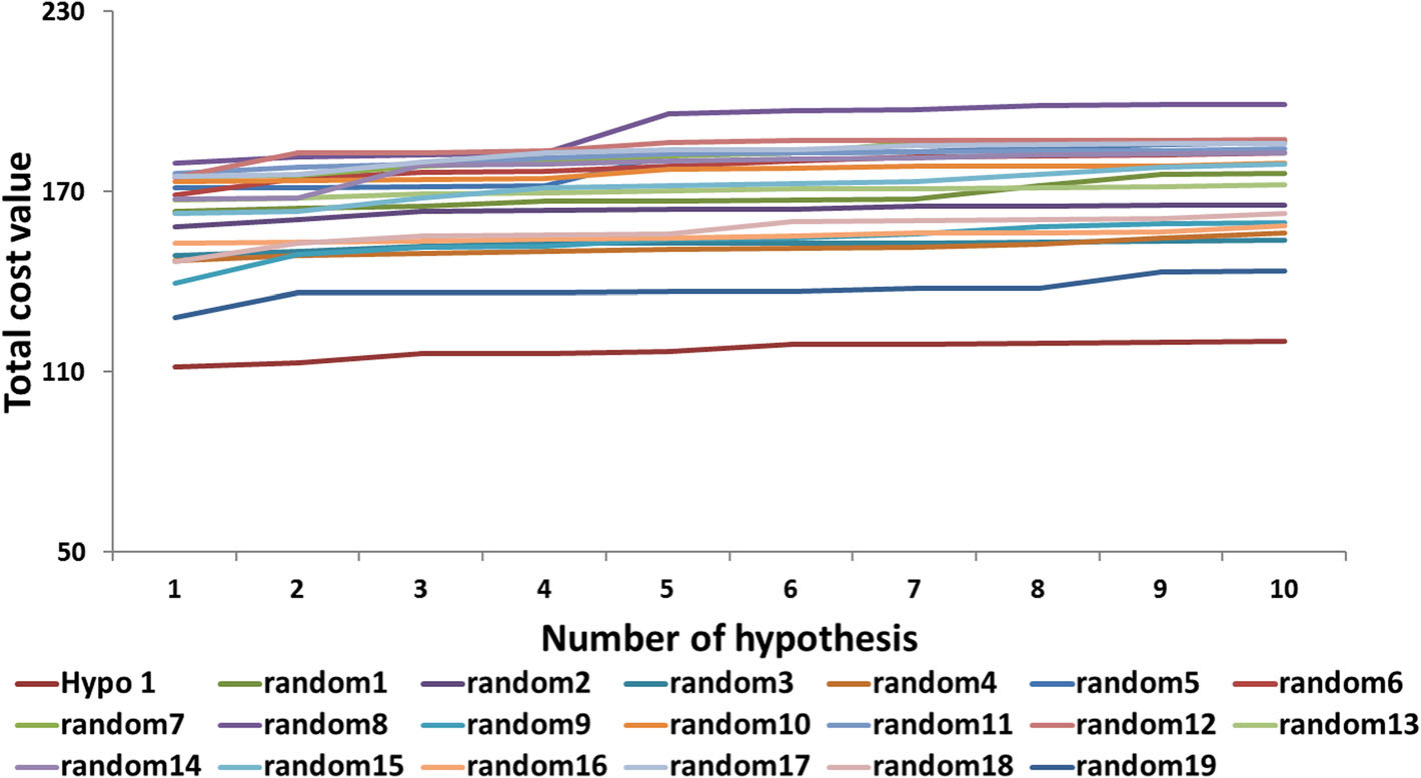
Validation by Fischer’s randomization method. Comparison of total cost of Hypo1 with the randomly generated 19 scrambles when 95% confidence level was selected

To ascertain the statistical robustness of Hypo1, Fischer’s randomization was performed that was run alongside the pharmacophore generation. A confidence level of 95% was selected which resulted in the formation of 19 spreadsheets. Thereafter, hypothesis significance was calculated employing the formula, [1-(1 + X)/Y] X100. Herein, X denotes sum of hypothesis, while Y indicates the total *HypoGen* runs, both the initial and the random runs. This method takes the advantage of assessing the correlation between the chemical structures and their corresponding biological activities, thereby quashing the chance correlation, and thus establishing that Hypo1 was not generated by chance. These results evidently state that the generated pharmacophore was of superior quality and thus, had least cost value as depicted in, Fig. 5.

#### Test set validation

**Fig. 6:**
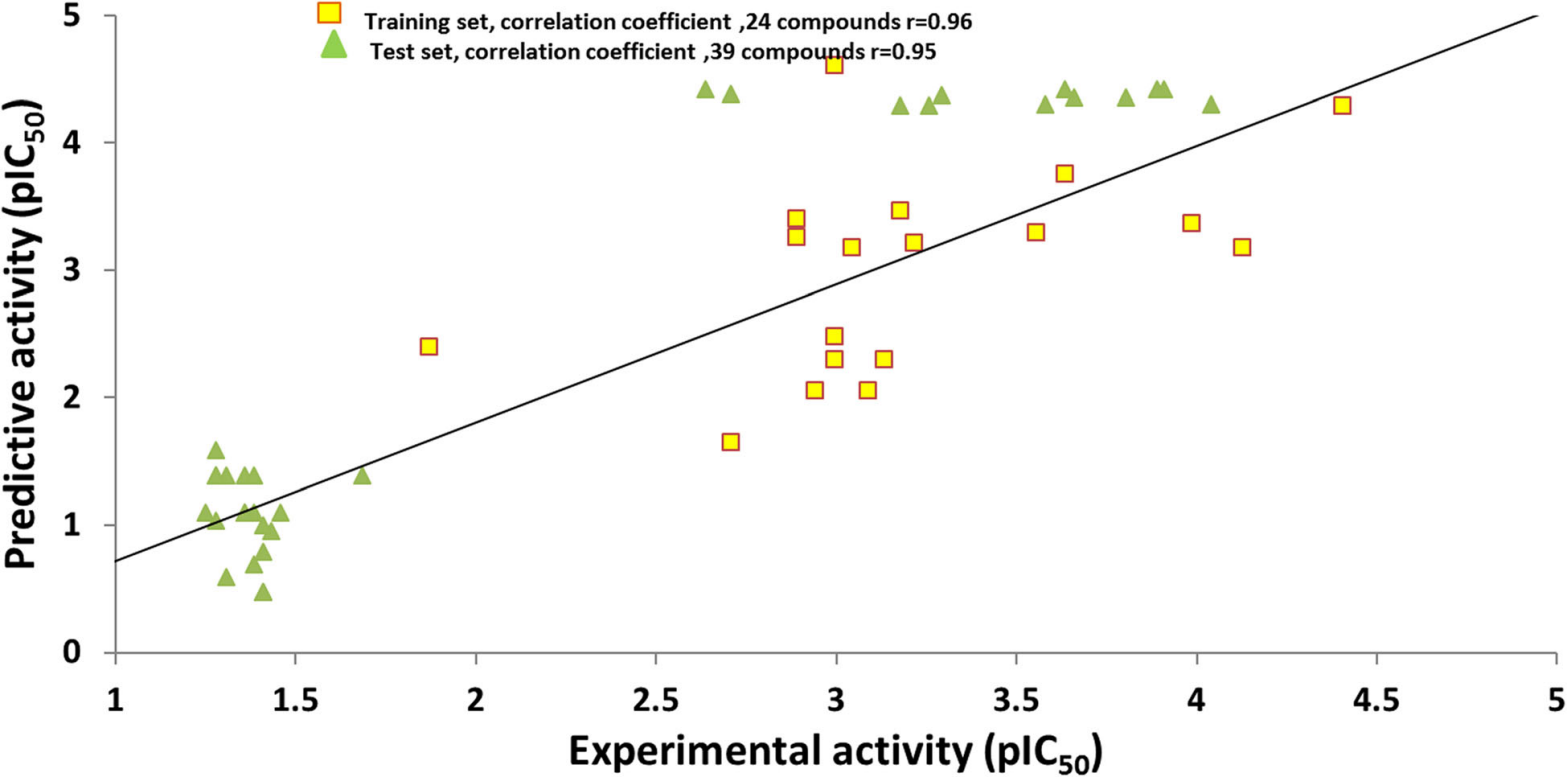
Correlation prediction of Hypo1 between the test set and the training set compounds

Test set method of validation was conducted to examine the ability of the Hypo1 in recognizing the compounds other than the training set and thus to classify them in the same order of the activity range. 39 structurally diverse compounds were collected and were grouped in accordance with the training set. Subsequently, the correlation coefficient (r) for the test set was computed as 0.95, while that of the training set compounds was 0.96, (Fig. 6). Additionally, Hypo1 ably estimated the activities of the external compounds, however, overestimated three moderately active compounds as active compounds, (Additional file 1). The test set validation results are a clear indicative of the fact that Hypo1 can be recruited to classify the external compounds as well.

#### Decoy set validation

To further quantify the usefulness of the Hypo1, it was subjected to yet another validation process, called the decoy set method. This method was executed recruiting an external database. Accordingly, a database (D) of 710 molecules was instituted with an addition of 15 actives (A). Database screening was performed and as a result 17 Hit compounds were retrieved (Ht) with 14 actives (Ha) in it. Further, the EF and the GF were computed to be 44.17 and 0.79 respectively, authenticating the goodness of the Hypo1, Table 3. Moreover, the percentage of ratio of the actives was found to be 93, pronouncing the high ability of Hypo1 in screening.

**Table 3:** Validation of Hypo1 by employing decoy set method

S. no	Parameters	Values
1	Total number of molecules in database (D)	710
2	Total number of actives in database (A)	15
3	Total number of hit molecules from the database (Ht)	17
4	Total number of active molecules in hit list (Ha)	14
5	% Yield of active [(Ha/Ht)	82
6	% Ratio of actives [(Ha/A) X 100]	93
7	Enrichment Factor (EF)	44.17
8	False negatives (A-Ha)	1
9	False Positives (Ht–Ha)	3
10	Goodness of fit score (GH)	0.79

^*^GH score between 0.6–0.8 is regarded as a good score

### Identifying the novel lead molecules through virtual screening

**Fig. 7:**
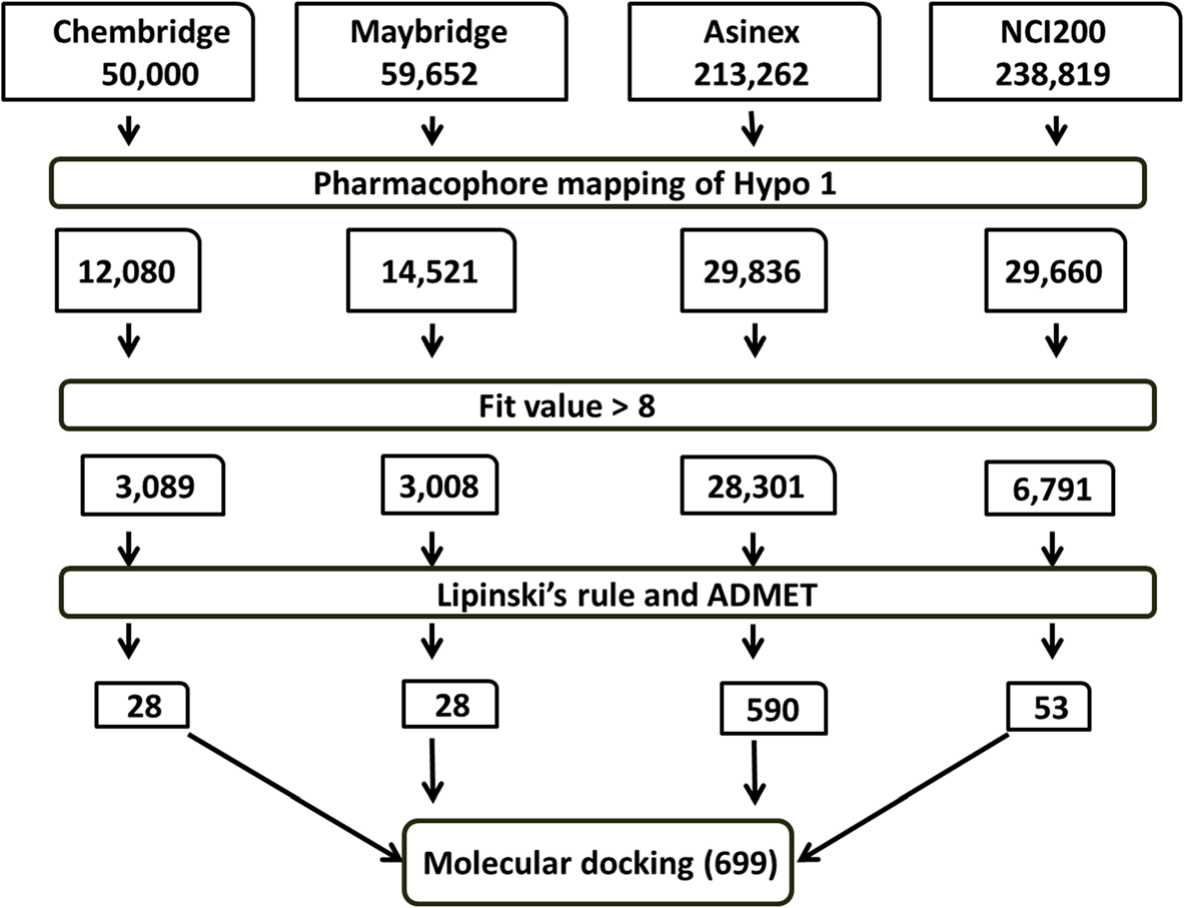
Schematic representation of methodology involved in virtual screening

Validated Hypo1 was employed to screen and retrieve the novel compounds against the VEGFR-2. The chemical features imbibed within the Hypo1 display an important role in identifying the new leads. Four databases, such as Chembridge, Maybridge, Asinex and NCI were selected for screening the dual inhibitors. Accordingly, Hypo1 has successfully mapped with 12,080, 14,521, 29,836 and 29,660 compounds from the aforementioned databases. Following which, compounds with a greater fit value than 8 were proceeded to the Lipinski’s Rule of Five and the ADMET studies. These two studies quantify the pharmacokinetics of a drug molecule and thus, is an essentiality for a drug molecule to qualify them. ADMET particularly computes BBB penetration, solubility, human intestinal absorption (HIA) solubility, plasma protein binding (PPB) and CytochromesP450 (CYPs) inhibition. The results were evaluated by setting the standard as 3, 3, and 0 for solubility, BBB and absorption, correspondingly. From the four databases, a total of 699 molecules were identified possessing the drug-like properties. The screened compounds along with the training set compounds were subjected to molecular docking analysis. The overall process of screening is represented pictorial form (Fig. 7).

### Molecular docking studies

Genetic Optimization for Ligand Docking, (GOLD) v5.2.2 was recruited for performing the molecular docking assay. GOLD has an ability to conduct lead optimization and to determine the accurate binding pose analysis. Technically, GOLD operates by two scoring functions, Goldscore fitness, and the Chemscore. Goldscore is a fitness parameter that is governed by four components, such as; protein-ligand hydrogen bond energy (external H-bond), protein-ligand van der Waals (vdw) energy (external vdw), ligand internal vdw energy (internal vdw), ligand torsional strain energy (internal torsion), respectively. This score was optimized for understanding and predicting the position of the bound ligand. On the other hand, Chemscore was used for rescoring and further computes the total free energy change on the ligand binding. The aptness of the docking parameters were assessed by subjecting the co-crystal to dock into the selected active site. This generated a reasonable RMSD of 0.9 Å between the docked pose and the co-crystal thus ensuring the accuracy of the GOLD parameters. Therefore, these parameters were considered for docking the screened compounds into the active site of the target, (Additional file 2).

Docking of the screened ligands and the training set compounds was initiated after specifying the radius of the active site around the co-crystal to 15 Angstrom (Å) followed by allowing the generation of 50 conformations for each ligand. All those compounds which fall in the selected radius were considered for the succeeding steps. The docking studies were initially executed with 4AG8, a potential drug target for angiogenesis. The final selected lead compounds after the DFT and the MD simulations were challenged with the CDK2 protein, 1URW. This strategy was conceived to identify a common drug molecule for both angiogenesis and progression.

**Fig. 8:**
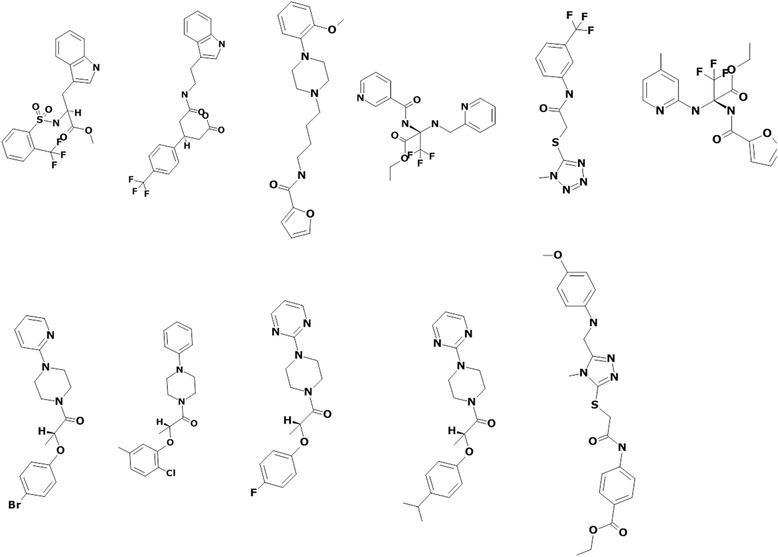
2D structures of final 11 compounds that comply to all the filters

The angiogenesis target for the present study was 4AG8, retrieved for the Protein Data Bank (PDB, http://www.rcsb.org/pdb/home/home.do). The reference molecule has displayed a dock score of 89.77 and therefore, only those lead molecules whose scores were above the reference molecules were chosen for further investigation and thus, qualifying 18 screened molecules. These compounds were examined manually for the hydrogen bond interactions with the amino acids residues, Cys919, Glu917, Asp1046, and Glu885, respectively. Out of 18 screened lead molecules, only 11 exhibited the hydrogen bond interactions with the key residues. These 11 compounds, (Fig. 8), obeyed all the filters employed to identify an efficient lead candidate. These 11 molecules were subjected to the Density Functional Theory (DFT) analysis for further understanding their molecular orbital energies.

### Density functional theory studies

**Fig. 9:**
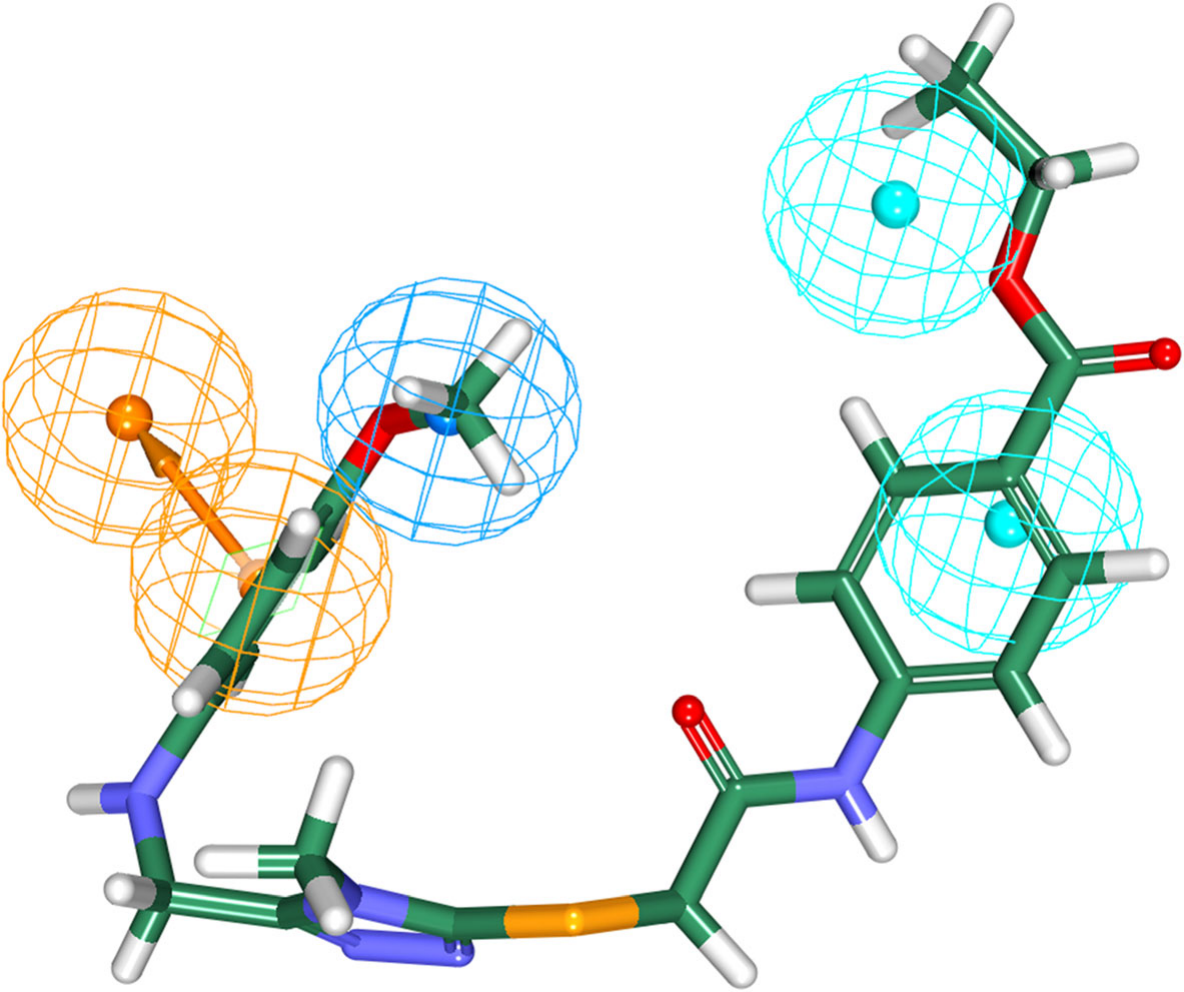
Mapping of the Hit compound to all the features of the pharmacophore. The compound is found to be well aligned with all the pharmacophore features

Highest occupied molecular orbital (HOMO) and the lowest unoccupied molecular orbital (LUMO) that ascribe the DFT were computed with six training set compounds (2 active, 2 moderately active and 2 inactive compounds) and 11 screened compounds. Small gap obtained, reports a compound to be highly reactive, while the larger gap signifies the presence of low reactivity of the compound to that of the target protein [62]. Accordingly, compound 4 has been selected as it demonstrated the least gap, Table 4, obeys the back to front approach and maps to all the pharmacophore features, (Fig. 9). Hereafter, is compound was named Hit.

**Table 4:** Calculation of the orbital energy values of Hit compounds and training set compounds utilizing DFT. Only top four candidates are tabulated

Name	HOMO (eV)	LUMO (eV)	∆E (eV)	IC_50_
Compound 1	−0.17	−0.02	0.14	
Compound 2	−0.18	−0.06	0.11	
Compound 3	−0.19	−0.07	0.12	
Compound 4(Hit)	−0.15	−0.08	0.07	
T1	−0.18	− 0.10	0.08	0.2
T2	−0.20	−0.11	0.09	5.2
T12	−0.18	− 0.07	0.10	26
T14	−0.18	−0.07	0.10	29
T23	−0.18	−0.09	0.08	41,000
T24	−0.20	−0.11	0.09	45,000

### Molecular dynamics simulations

**Fig. 10:**
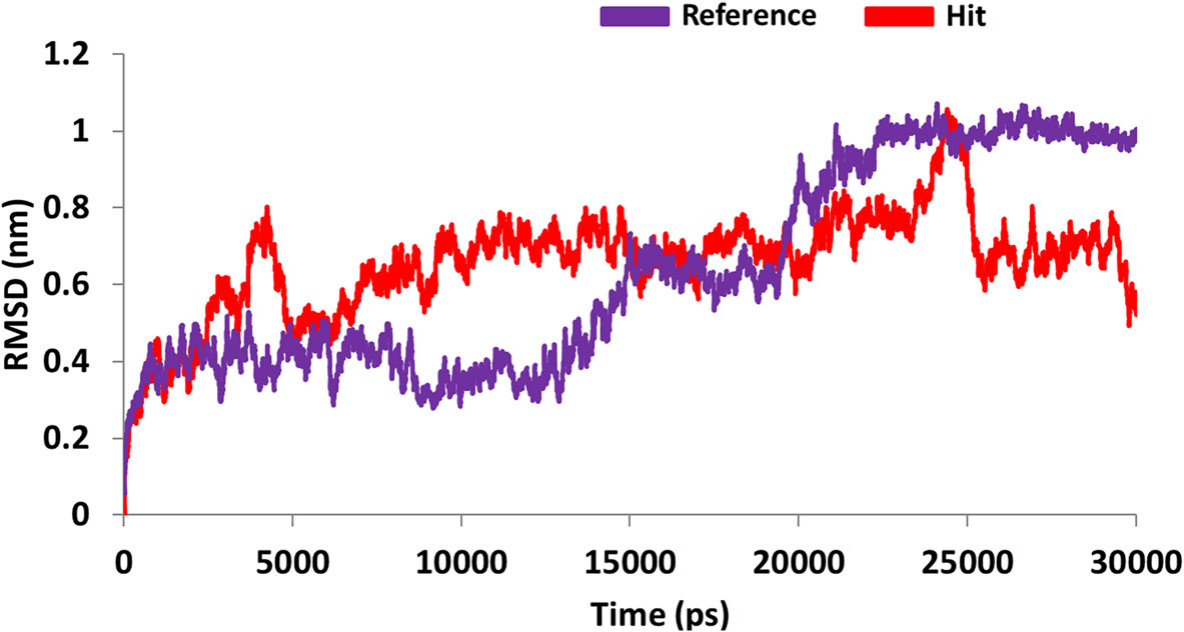
Quantifying the overall stability of the backbone during 30 ps. Purlpe denotes the protein-reference complex and red denotes the protein-Hit complex

**Fig. 11:**
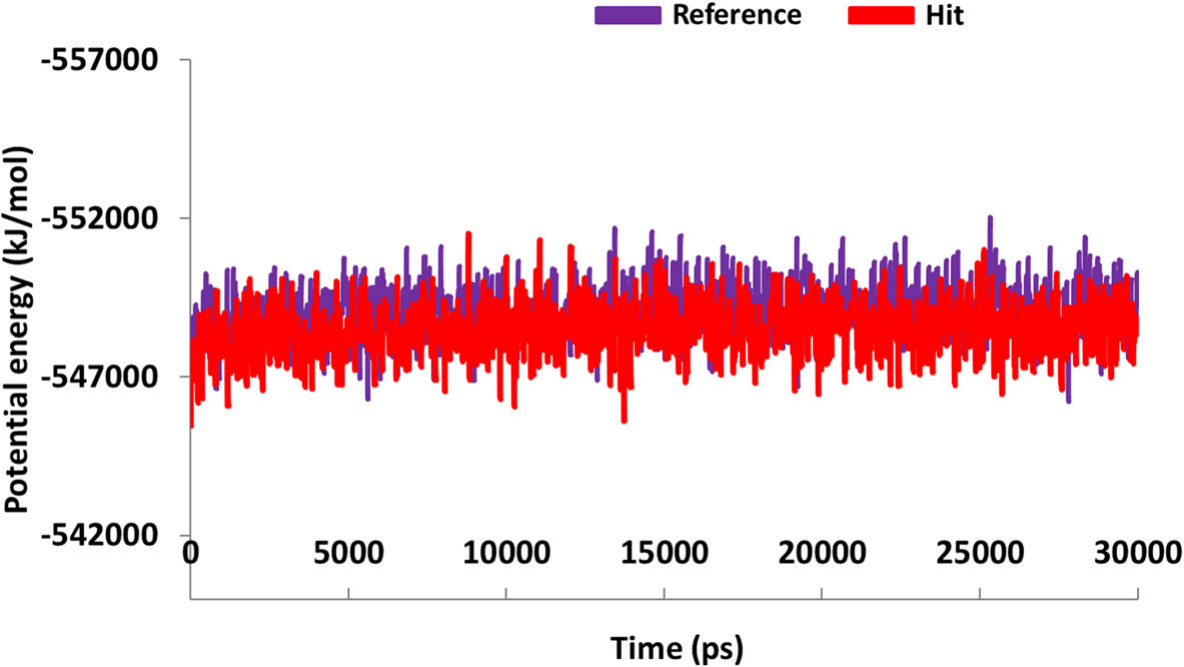
Potential energy of protein-reference (purple) complex and protein-Hit complex (red). The plots show that both the complexes were well converged between − 547,000 kJ/mol and −551,000 kJ/mol

**Fig. 12:**
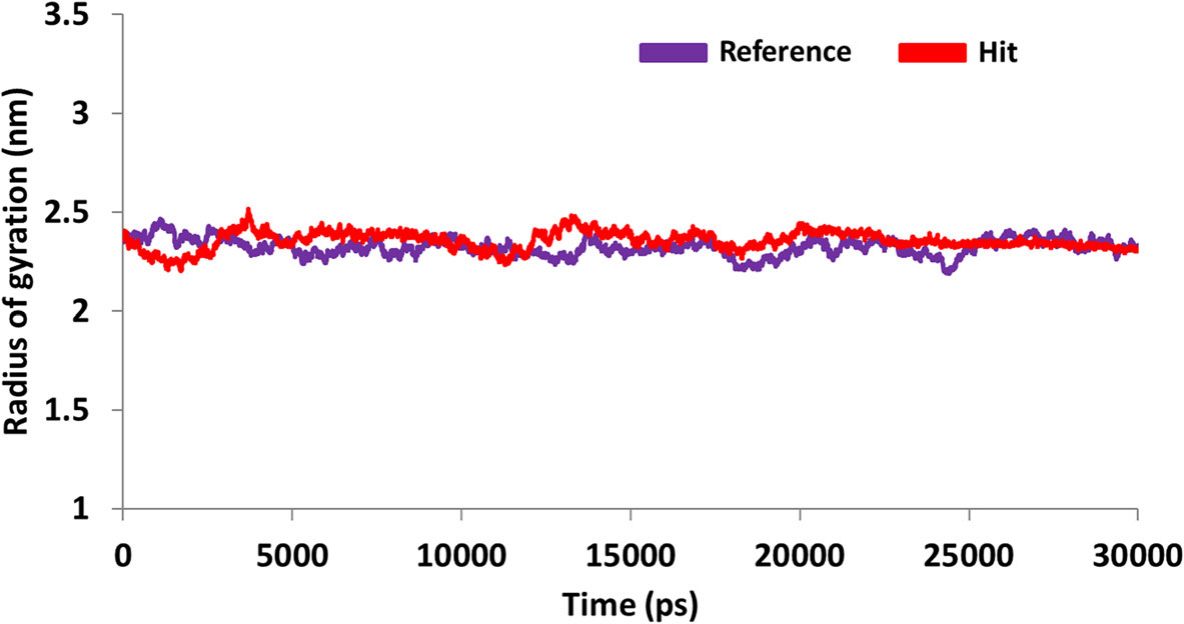
Radius of gyration profiles protein-reference (purple) complex and protein-Hit complex (red). The protein-Hit complex was observed to be stably compact than the reference

With an objective of assessing the binding stability of the final systems, the best conformations obtained from the docking were proceeded to MD simulations. Furthermore, MD simulation evaluates and delineates on their dynamic behaviour and with each other. The MD results were examined for the RMSD values, potential energy and the radius of gyration to assess the stability of the protein backbone. The RMSD values were found to be 1.0 nm, and 0.8 nm, respectively for the reference molecule and the Hit. Additionally, it can be observed that the RMSD profiles of the candidate compound was more stable than the reference, (Fig. 10). It was also noticed that the stability was attained after 20 ps for reference compound and while Hit 1 was stable after 25 ps, (Fig. 10). Furthermore, their stability was assessed by plotting the potential energy, (Fig. 11) and the radius of gyration, (Fig. 12). Both profiles have rendered results that showed no abnormal behaviour throughout the simulation. Furthermore, the protein-Hit complex was found to be more compact as compared with the protein-reference complex, (Fig. 12). Additionally, from the Fig. 12 it can be understood that there were a few aberrations exhibited by the protein- reference complex and was stable after 25 ns. On the contrary, the protein-Hit complex was found to be stable after 16 ns, showing no aberrations thereafter.

**Fig. 13:**
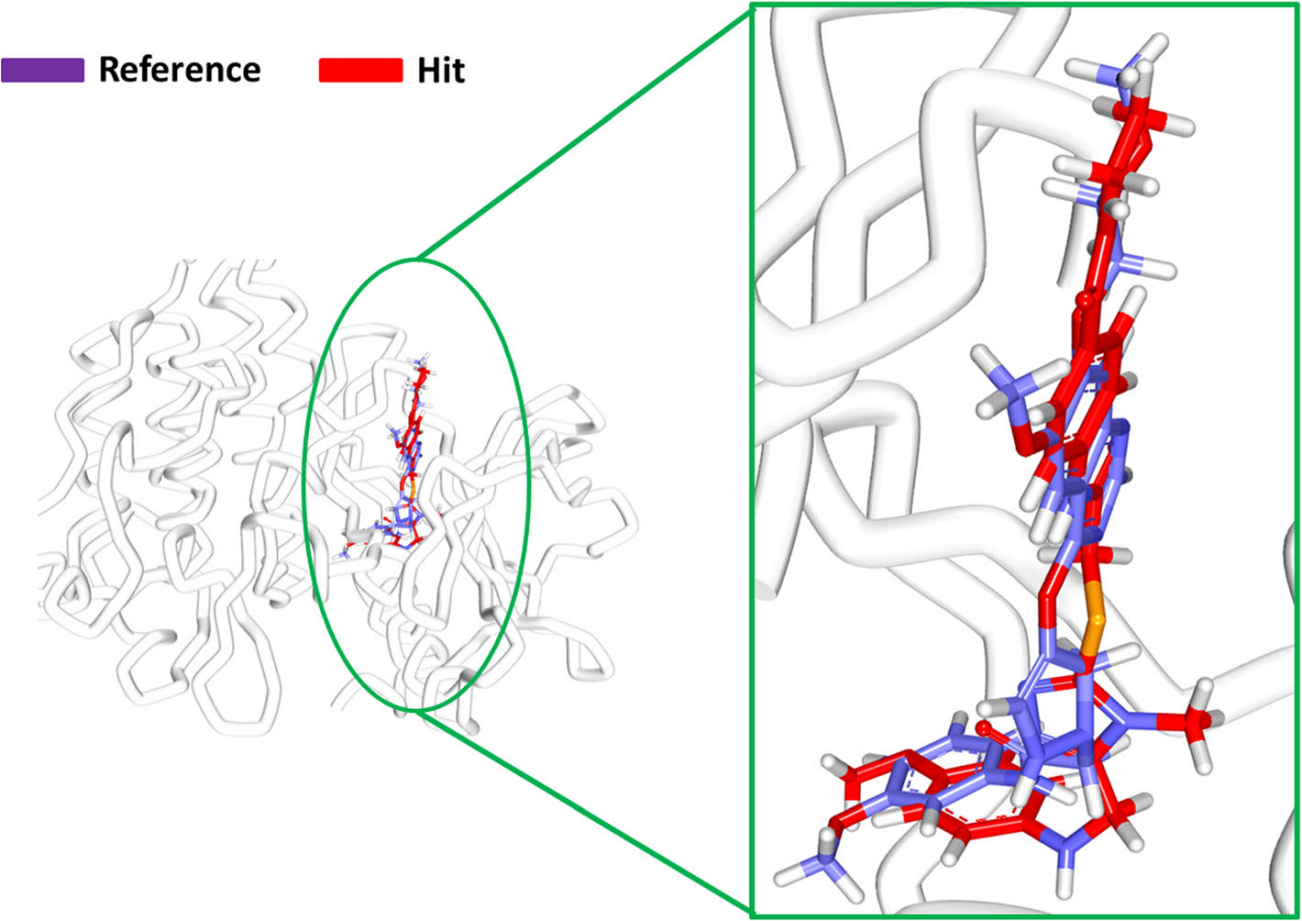
Assessment of the binding mode patterns of the reference (purple) and the Hit (red). The Hit obeys the same binding pattern of the reference compound

**Fig. 14:**
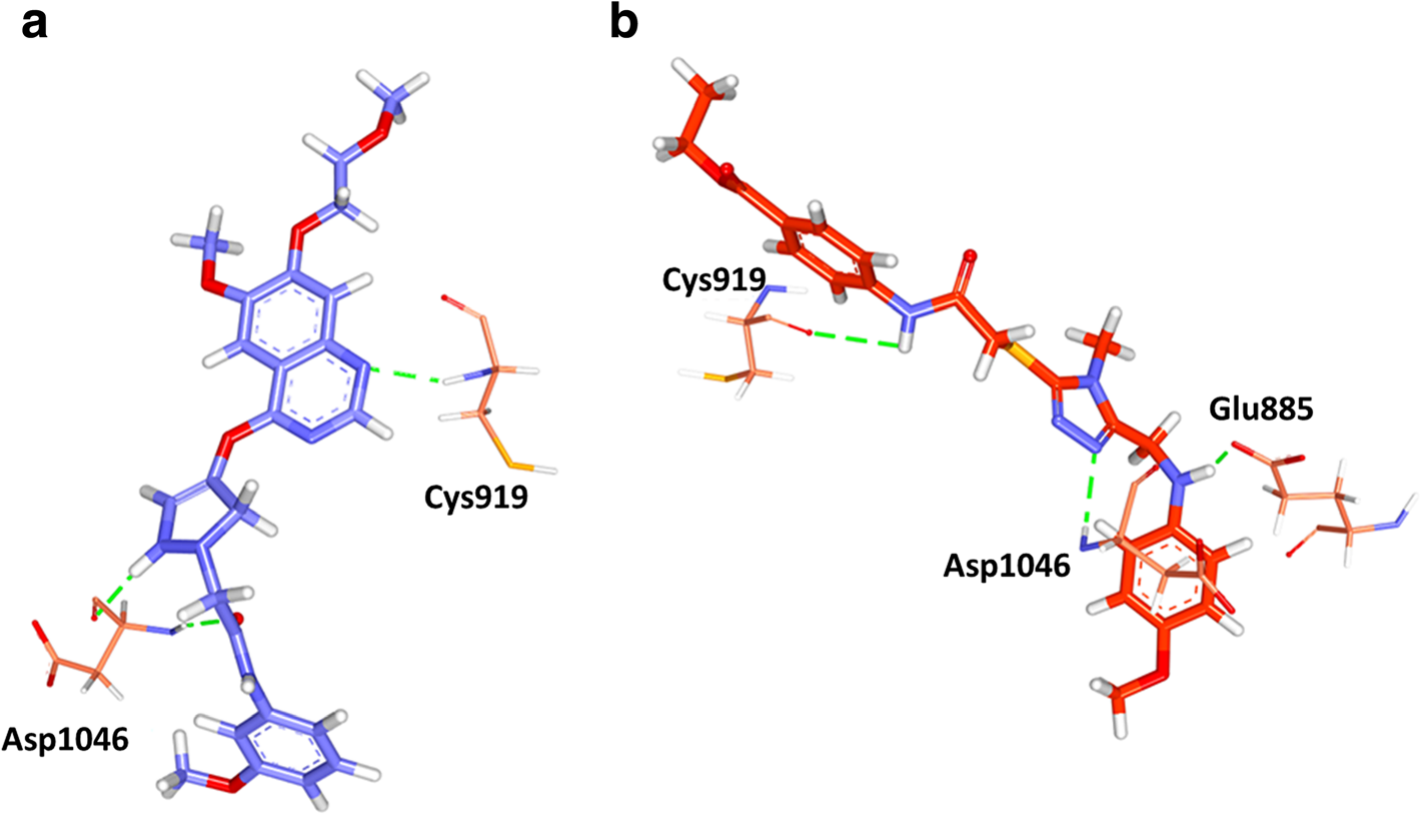
Molecular interaction between the reference- protein (purple) and Hit-protein (red). Green dotted lines indicate the hydrogen bonds. The residues are represented in orange stick model

**Fig. 15:**
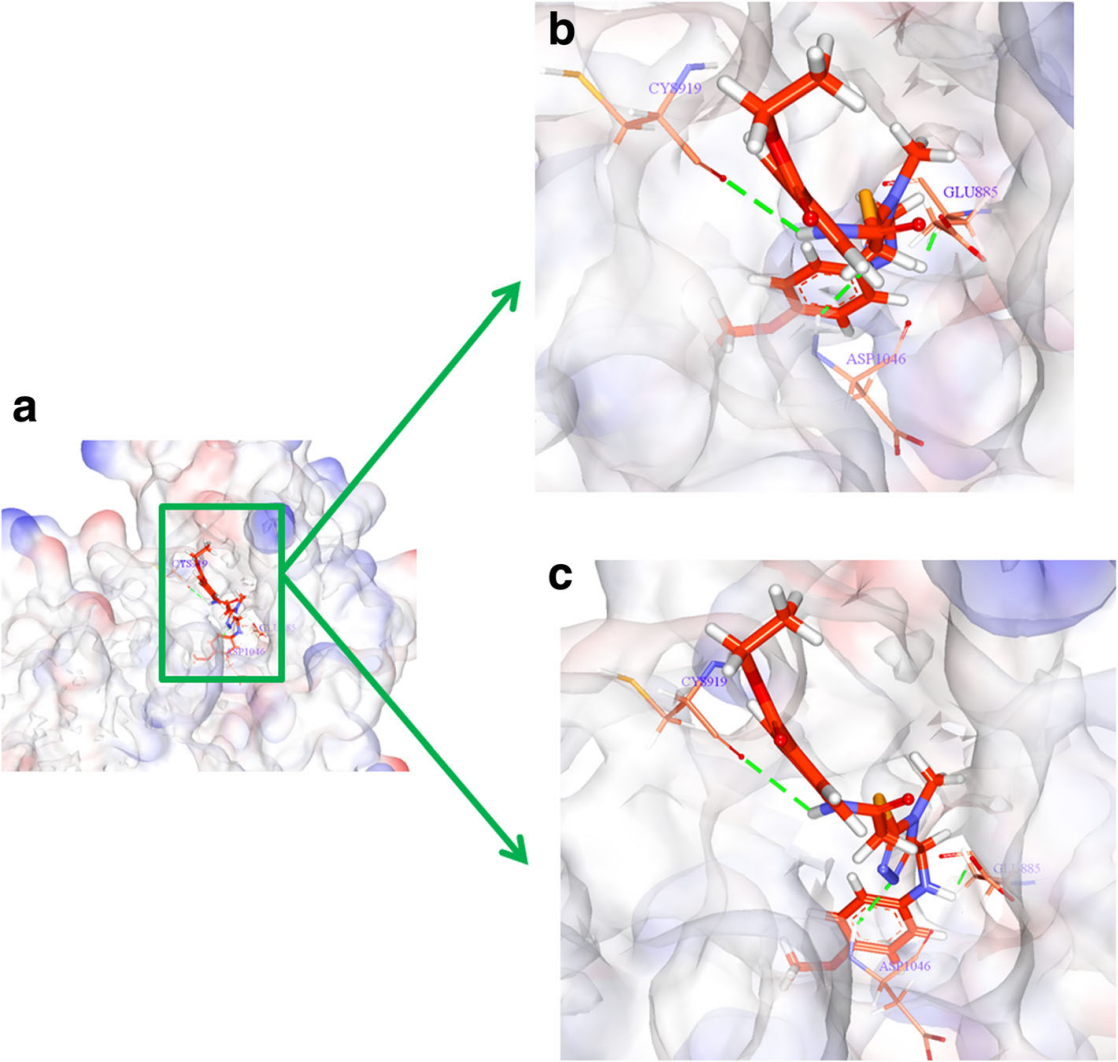
Back-to-front pattern of binding. a) Depiction of the presence of Hit in the active site pocket in back-to-front fashion. A clear enlarged cavity is represented in b and c. The ligand was found to be seated firmly with Cys919 from the top and is sandwiched with Glu885 and Asp1046

**Fig. 16:**
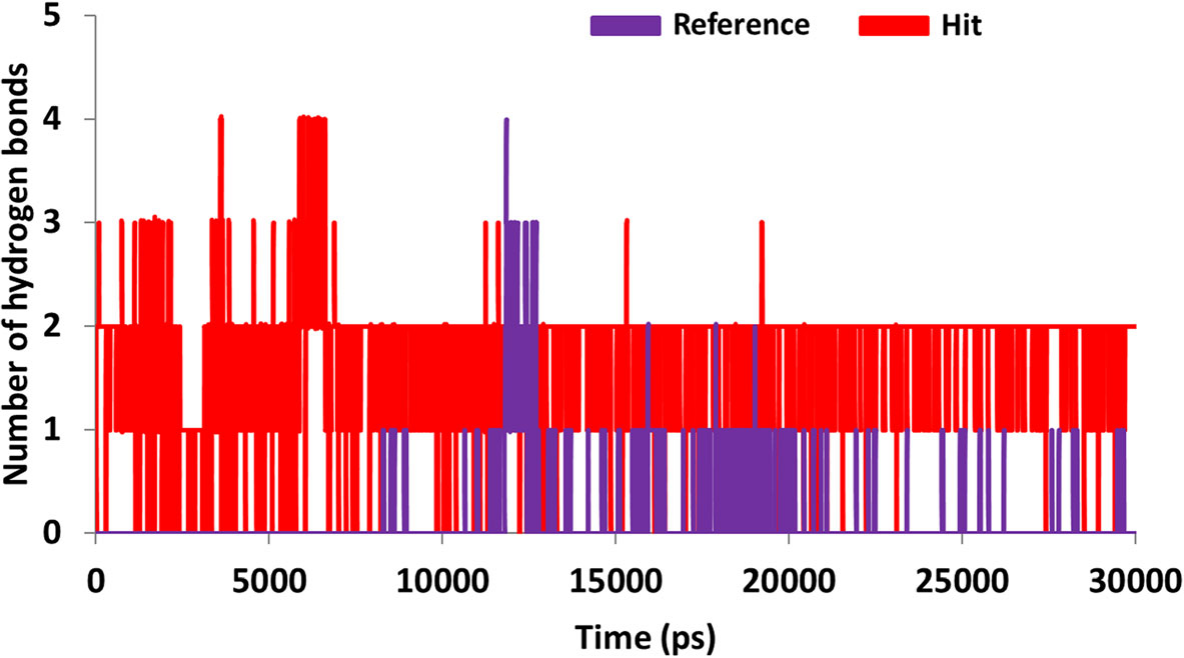
Estimation of the hydrogen bond interactions during the whole simulations. The refrence is represented in purple and red denotes the Hit. The reference has produced lower number of hydrogen bonds and predominantly seen during 11,000 ps ~ 20,000 ps. The Hit has shown regular bonds throughout the simulations

The binding mode analysis was performed retrieving the representative structures from the last 3 ns trajectories. Upon superimposition, it was observed that the binding pattern of the Hit was similar with the reference compound, (Fig. 13). Further inspecting the molecular interactions of the reference, it was revealed that the reference has formed three hydrogen bonds demonstrated by two key residues, Cys919 and Asp1046, respectively and displaying an acceptable bond length, Table 5. The HN atom of the Cys919 interacted with N7 atom of the ligand and NH and O atoms of Asp1046 have joined with O26 and H62 of the ligand, (Fig. 14a). Leu840 has participated in the π-sigma interaction, while the residues, Ala866, Ala866, Val898, Leu889, and Leu1035 were found to interact through the π-alkyl bond, (Additional file 3). The Hit has interacted with the protein target with three hydrogen bonds represented one each by Glu885, Cys919 and Asp1046, respectively, (Fig. 14b). HN atom of Cys919 has involved with the ligand’s O14 atom with a hydrogen bond length of 2.16 Å and the OE2 atom of Glu885 joined to the OE2 atom of the ligand displaying a bond length of 2.09 Å, respectively. Additionally, Leu840, Val848, Lys868, Leu1035 have interacted involving with the π-π bonds. Leu889 and Phe918 have interacted with the π-alkyl bond and thus make the ligand to be seated firmly within the active site groove, (Additional file 4). Moreover, from the large set of screened databases only one Hit was identified has the only retrieved compound that obeyed back-to-front pattern of binding and thus projecting itself as a unique lead compound, (Fig. 15). Correspondingly, it was observed that the Hit molecule has been positioned within the DFG motif comprising of Asp1046-Phe1047-Gly1048 residues sandwiched between Asp1046 and Glu885 on either side and hence crucial in conferring the reduction of angiogenesis. The Cys1045 has formed a π- sulfur bond that makes the ligand to be placed firmly with the active site. A hydrophobic interaction have additionally concentrated towards the DFG motif of the activation loop and thereby, distorts the allosteric change of the motif and inhibits the kinase activity [5] and therefore the Hit may act as type II inhibitor. Further details of the interactions are tabulated in Table 5. Furthermore, to gain insight into and attain a proper reasoning on the nature of the hydrogen bonds in the active site, the hydrogen bonds were recorded throughout the simulation time. Consequently, it was noticed that the reference has produced an average of 0.7 hydrogen bonds, while the Hit has displayed a higher average of 1.7, (Fig. 16).

**Table 5:** Intermolecular interactions between the VEGFR-2 inhibitors

Name of the compound	Hydrogen bonds < 3 Å			van der Waals interactions	π-π
	Residue atom	Ligand atom	Bond length		
Reference	Cys919:HN Asp1046:NH Asp1046: O	N7 O26 H62	2.2 2.0 2.0	Val848, Lys868, Glu885, Ile892, asn900, Leu901, Lys920, Phe921, Gly922, Asn923, Leu1019, leu1044, His1026 Phe1047	Leu840, Ala866, Ala866, Val898, Leu889, Leu1035
Hit	Glu885:OE2 Cys919: O Asp1046:HN	H36 H35 N5	1.88 2.9 2.3	Ala866, Glu850, Ile888, Ile892, Val898, Val914, Lys920, Asn923, Gly922, Leu1019, His1026, Ile1044, Phe1047	Leu840, Val848, Leu889, Lys868, Phe918, Leu1035

### Challenging the identified lead molecule against CDK2 protein

After achieving the first objective, now the investigation proceeds to understand if the ligand is potential enough to act against cancer prognosis. Cyclin-Dependent Kinase 2(CDK2) has been attributed with certain cancer progressions, especially the oral cancer [63, 64]. Progression refers to the advancement of the disease during the course of the time. For the current study the protein 1URW, was retrieved from the protein data bank. This protein is in complex with imidazole [1,2-B] pyridazine inhibitor of 1.6 Å. The identified lead molecule form the above steps is now challenged with 1URW, such that a common drug could be identified.

**Fig. 17:**
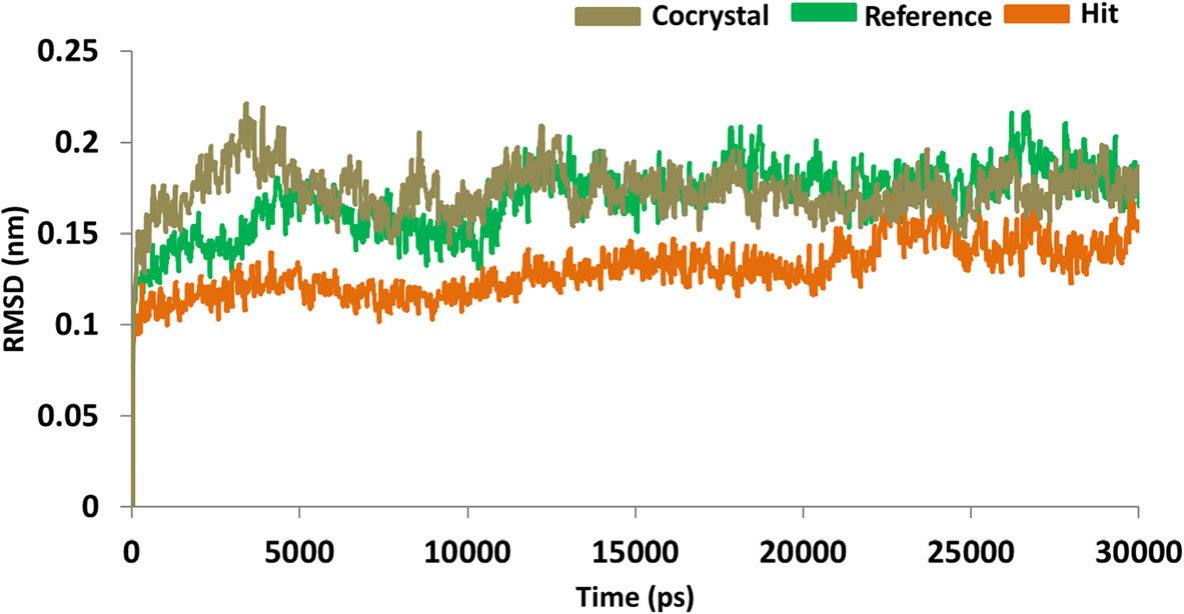
The RMSD backbone stability of the three systems during 30 ns run

All the steps, such as, protein preparation and ligand preparation were followed as per the aforementioned methods. The active site of the protein was plotted at 12 Å around the inbuilt co-crystal. To ensure the suitability of the docking parameters, the co-crystal was initially docked and an acceptable RMSD of 1.75 Å was generated, (Additional file 5) and the same parameters were considered for docking employing GOLD v5.2.2. The results were examined taking into consideration that the inhibitor lies within the selected active site sphere and forms a hydrogen bond with Leu83, Asp86 and Lys89. Amongst them, Leu83 is the core residue as reported earlier [26, 65]. To authenticate the binding of the ligand with the active site residues, the results generated by the co-crystal docking were taken into consideration. The best pose generated was escalated to MD simulation studies to assess the protein backbone stability and were read as root mean square deviation (RMSD). Subsequent results demonstrated that the cocrystal, reference and the Hit have displayed stable RMSD values that exist below 0.25 nm throughout 30 ns run, (Fig. 17).

**Fig. 18:**
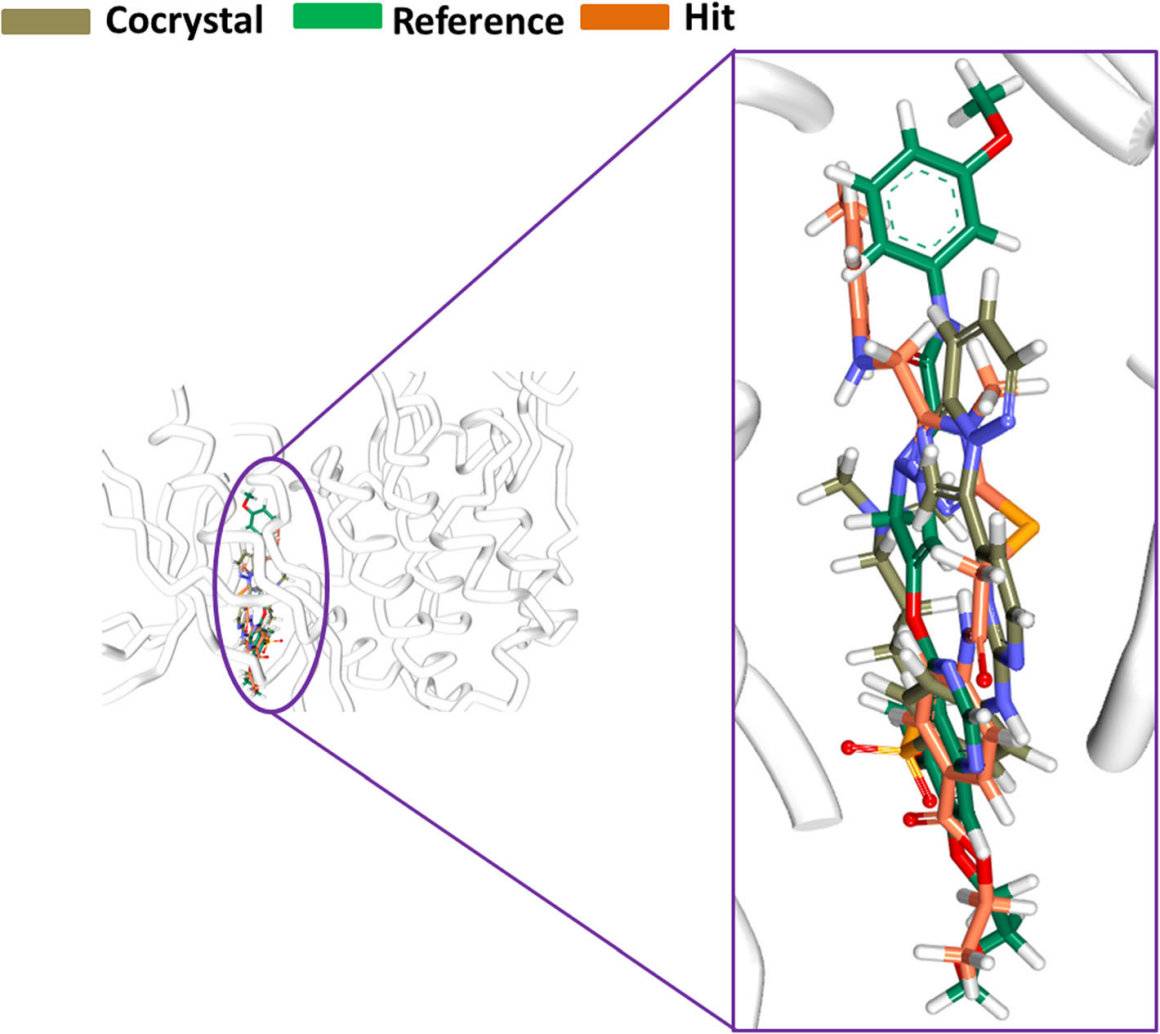
Binding mode assessment of compounds. The co-crystal is represented in gray, reference is denoted in green and the Hit in orange. All the three follow the same pattern

**Fig. 19:**
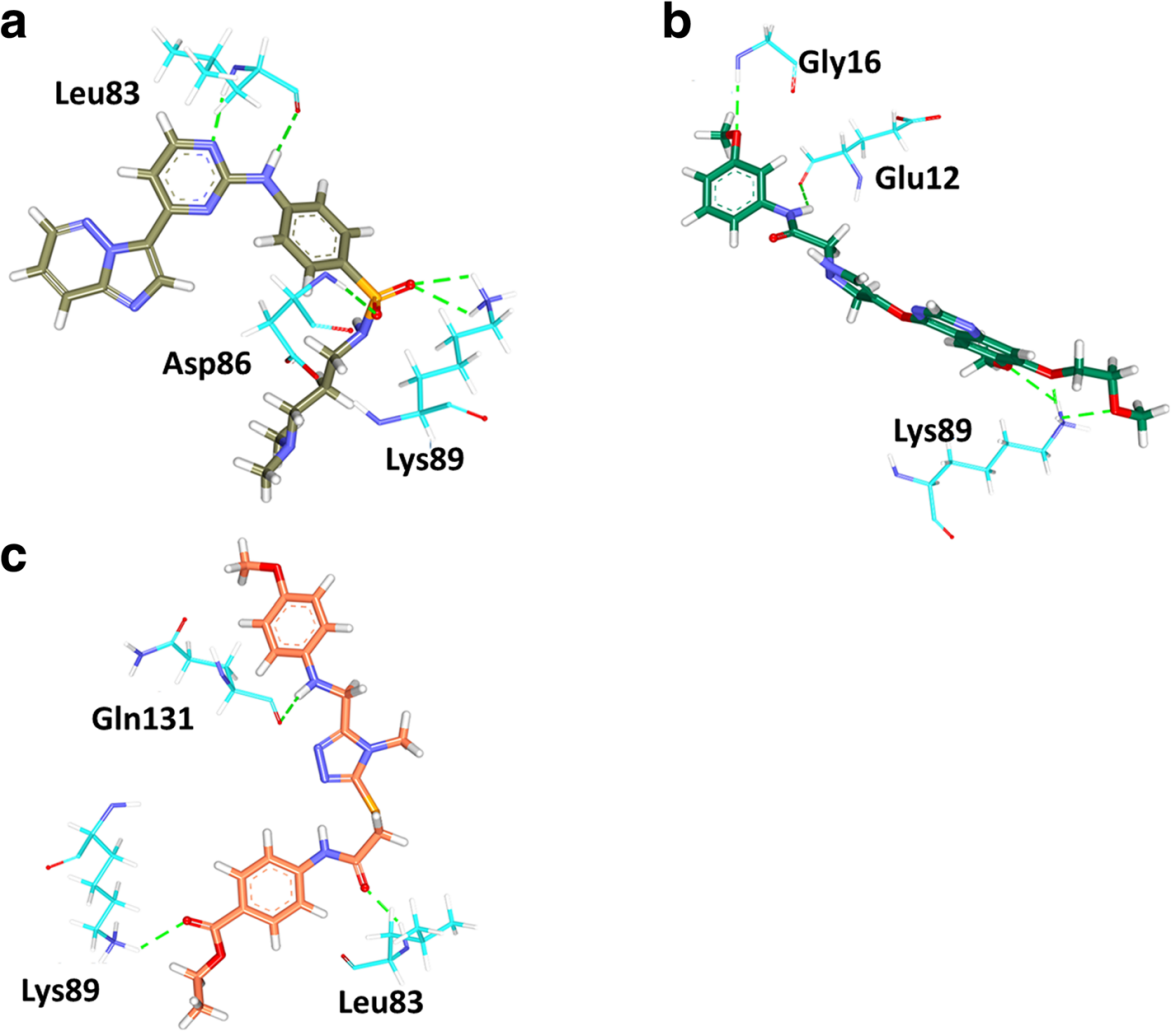
Intermolecular interactions between the ligand and the protein. The co-crystal is represented in gray, reference is denoted in green and the Hit in orange. Green dotted lines represent the hydrogen bonds. The protein residues are indicated in cyan

The representative structures from the last 3 ns were extracted and superimposed for further analysis. Upon superimposition of the co-crystal, reference and the Hit have demonstrated a similar type of binding mode, (Fig. 18). The dock results revealed that the co-crystal has formed four hydrogen bonds with residues namely Leu83, Asp86 and Lys89 demonstrating a dock score of 53.05, while the reference and Hit has displayed a score of 52.56 and 64.34. Additionally, a number of hydrophobic bonds have been generated in the form of van der Waals interactions and π-π bonds as represented in Fig. 19a and Additional file 6. The reference compound on the other hand has rendered five hydrogen bonds one each by Glu12 and Gly16 and three bonds with Lys89. It was observed that only the Lys89 residue was noticed to participate as was seen in the crystal structure. Additionally, Glu12 formed a weak hydrogen bond of 3.0 Å and displayed no interaction with Leu83. This could be because the compound is a specific VEGFR-2 inhibitor. However, it exerts its effect through the involvement of Lys89 residue, (Fig. 19b: Additional file 7). The retrieved Hit has displayed three hydrogen bonds involving two crucial residues, Leu83 and Lys89 and further has generated an acceptable bond length of 2.1 Å and 2.4 Å, respectively, Fig. 19C. Additionally, it formed more number of hydrophobic interactions with the key resides, (Additional file 8). The details of the molecular interactions are tabulated in Table 6.

**Table 6:** Intermolecular interaction between the protein and the ligands

Name of the compound	Hydrogen bonds < 3 Å			van der Waals interactions	π-π
	Residue atom	Ligand atom	Bond length		
Co-crystal	Leu83: O Leu83:HN Asp86:HN Lys89:HZ2 Lys89:HZ3	H42 N20 O31 O32 O32	2.2 2.0 2.5 2.4 2.4	Gly11, Glu12, Lys20, Lys33, Val64, Glu81, Gln85, Lys88, Gln131, Asn132, Leu148,	Val18, Ala31, Phe80, Phe82, Leu134, Ala144
Reference	Glu12: O Gly16:HN Lys89:HZ2 Lys89:HZ2 Lys89:HZ3	H43 O27 O34 O31 O29	3.0 2.2 2.3 1.9 2.5	Gly11, Tyr15, Val18, Gln85, Asp86, Asp127, Lys129, Gln131, Asn132, Leu134, Leu298,	Phe82
Hit	Leu83:HN Lys89:HZ2 Gln131: O	O25 O24 H36	2.1 2.4 2.2	Gly11, Glu12, Ala31, Lys33, Val64, Phe82, His84, Gln85, Asp86, Lys129, Asn132, Asp145, Ala144, Val164	Ile10, Val18, Leu134, Leu298

**Fig. 20:**
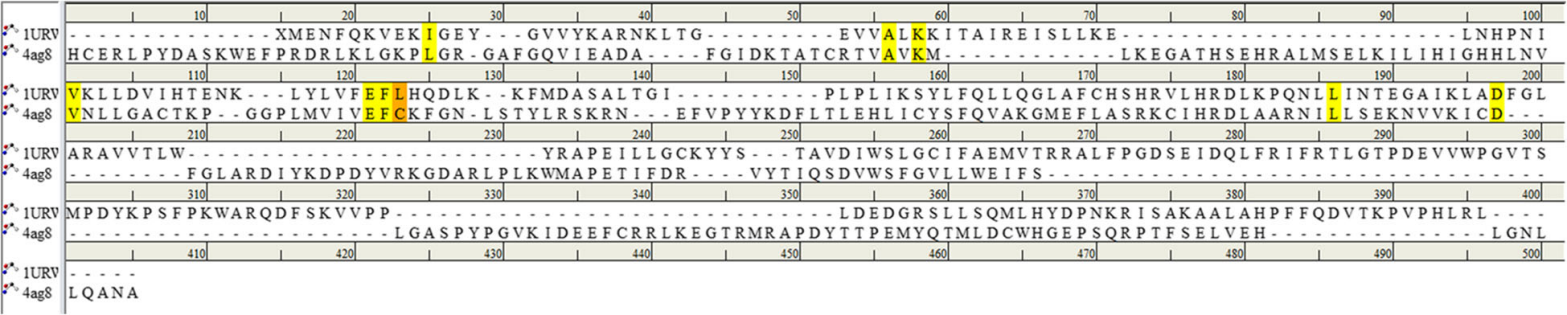
Sequence alignment of the protein targets representing angiogenesis (4AG8) and progression (1URW). The sequence identity and the sequence similarity were found to be 20.1 and 38.1, respectively. The active site shows higher degree of identity as demonstrated by the residues highlighted in yellow. The orange amino acids refer to the key inhibitory residues found to be complementary with each other

**Fig. 21:**
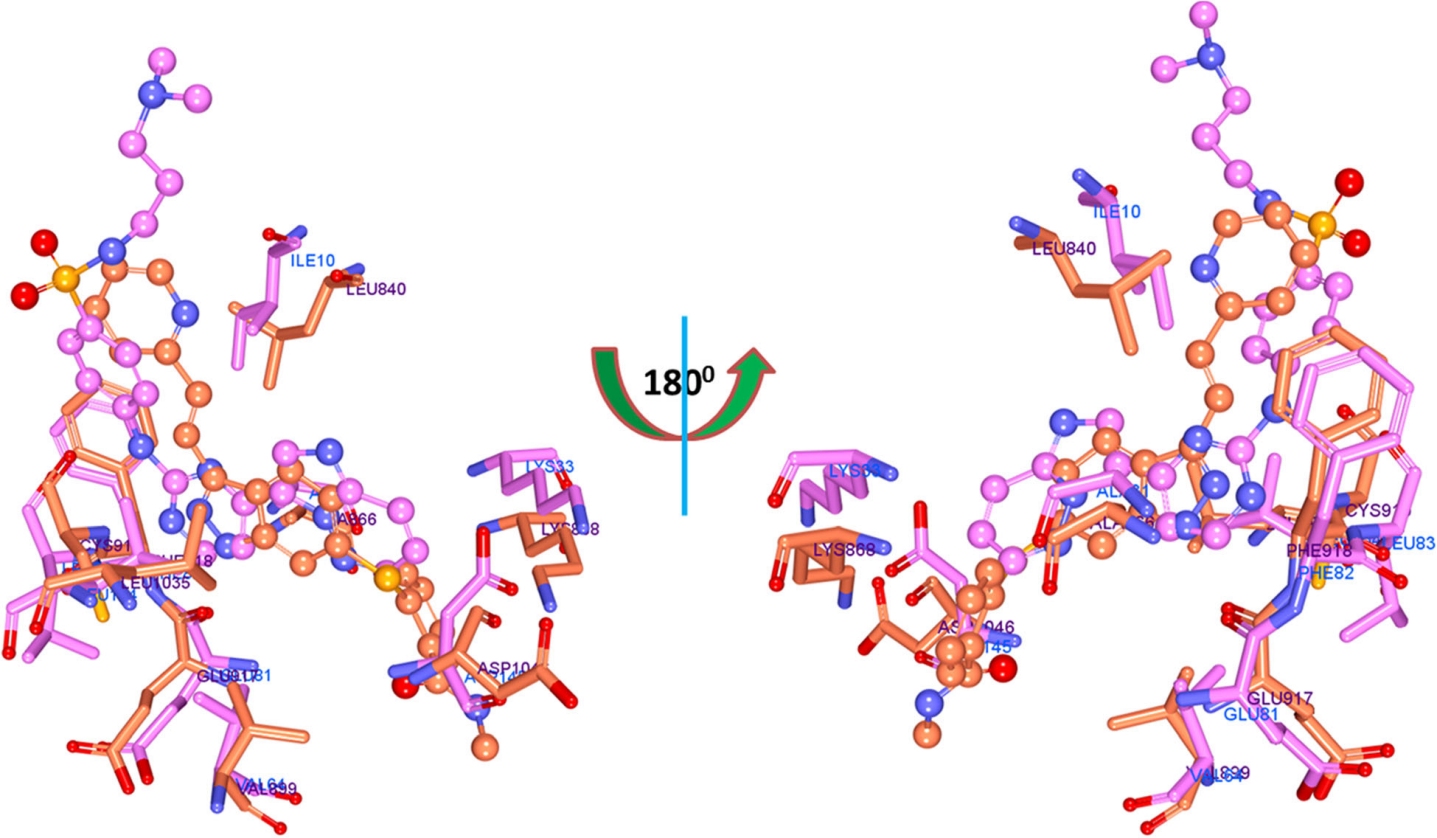
Comparison of the active sites of 4AG8 (orange) and 1URW (pink). The corresponding ligand molecule is represented in ball and stick model

Furthermore, the binding pockets of both the targets were evaluated to understand their similarity and was assessed using PocketMatch (PM) [66] that was executed through three aspects (a) representation of each site as sorted lists of distances between chosen points, (b) alignment of two sets of distance lists and (c) choosing a scoring scheme for arriving at a final score. The similarity is read as PM score which ranges from 0 to 1, where zero implies no similarity and 1 refers to complete similarity. The obtained results revealed the PM score to be 0.82 (0.6 or greater refers to almost similar). To further affirm the similarity of the active sites, the *align structures* protocol available on the DS was initiated and subsequently, the arrangement of the sequences, (Fig. 20) and the active site residues, (Fig. 21: Additional file 9) were evaluated. It was interesting to note that the majority of the key residues were conserved besides; the key residues Cys919 and Leu83 were complementary with each other and were instrumental in rendering the inhibitory activity in the corresponding drug targets. These findings further reinforce that they share similar binding pocket facilitating the use of common drug.

## Discussion

Receptor tyrosine kinases (RTKs) are a class of proteins that play a crucial role in cell proliferation, differentiation, metabolism and cell growth and survival. Among them, vascular endothelial growth factor receptor-2 (VEGFR-2) is important in angiogenic regulation and hence is a major target for repressing the cancer growth and metastasis [67–70] . Additionally, the uncontrolled development of these cancerous cells is the main reason for metastasis. Therefore, in the current study we aimed at identifying a small molecule from the large datasets that could selectively inhibit both the process, thus leading to suppression of cancer. Accordingly, only one lead candidate has been identified that binds in back-to-front approach with the VEGFR-2 and also inhibits the CDK2. The identified inhibitor successfully binds to the ATP site (front pocket) and also extends towards the hydrophobic site (back pocket) and hence categorizes itself has type -II inhibitor. Cys919 located at the front pocket holds firmly the ligand molecule by HN atom and facilitates the bulky benzene ring to be accommodated within the active site with a slight tilt formed at the tip of the ligand comprised of CH_3_ group, (Fig. 15b). The back pocket on the other end was observed to be held tightly with two hydrogen bonds and several hydrophobic bonds, (Fig. 15c: Additional file 4). The pentane ring of the ligand that is located at the ligands center was seen to be held with two hydrophobic bonds produced by Val848 and Lys868 besides displaying a hydrogen bond with Asp1046. Therefore, the presence pentene ring in the ligand apparently seems to be important in making this inhibitor of a type II class. Additionally, the sulphur group of Cys1045 has involved with the pentane ring. Additionally, the first benzene ring was held by Leu840 and Leu1035 and the second benzene ring was held by Leu889. Furthermore, it can be observed that the bulky groups present within the ligand have been held by either hydrogen bonds or the hydrophobic bonds, thus providing proper positioning for the ligand.

Furthermore, we challenged the identified Hit against CDK2 target whose inhibition can abrupt the progression of the cancer cells. The CDK2 displays four binding sites such as ATP binding site (competitive) and two non- competitive sites. Additionally, upon subjecting to the cyclin binding process gives rise to a new site caused due to the conformational changes in the ATP site and is labeled as the allosteric site (site IV). Amongst them the ATP binding site is highly conserved across all the human kinases [23] . Therefore, we focused on the ATP binding site to evaluate the potency of the identified ligand. This ATP binding site is a cleft located between the N-terminal domain consisting of beta-sheets (small lobe) and a C-terminal domain rich in helices (large lobe) [71], which can be further divided into three. The first one is the hydrophobic region comprising of Ile10, Ala31, Val64, Phe80, Glu81, Phe82, Leu83, Leu134 and Ala144. The second region consists of three amino acids, Val18, Asp86 and Gln131 and generally interacts with the ribose moiety of the ATP. The third region is made up of Lys33, Asp145 and Asn132. Additionally, two domains are joined by the hinge region constituting Glu81 and Leu83, respectively. The identified ligand has formed an hydrogen bond interactions with the key residues involving the Leu83 and Lys89 [65, 71]. An additional hydrogen bond was formed with the amino acid Gln131 that is located at the polar interaction site through its HN group. This pattern of binding is in agreement as described previously [71]. The residues belonging to the hydrophobic region consisting of Ile10, Val18 and Leu134 have joined with the ligand by the hydrophobic bonds. The residues, Val64, Ala144 of the hydrophobic interaction site and Lys33, Asp86 and Asp145 that belong to the polar interaction site have interacted with the ligand through the van der Waal’s interactions and thus facilitating the lead molecule to be seated firmly with in the active site. Furthermore, since the ATP binding sites are conserved across the kinases [71], this could be one reason for the complementarity that exists between Cyc919 of VEGFR2 and Leu83 of CDK2, (Fig. 20).

## Conclusions

The present study aims at identifying a dual inhibitor that can repress the angiogenesis and progression. In order to achieve this, a systematic ligand-based pharmacophore modelling and subsequent large database screening was conducted. From the obtained lead molecules, further knowledge based screening was conducted to find the compounds that obeyed the back-to-front type of inhibitory mechanism employing the docking protocol and the interactions with the key residues. Consequently, results showed that only one compound has qualified this criterion. Upon further studies and applying the MD simulations, it was observed that the ligand-protein complex was relatively stable. Therefore this lead compound was challenged against 1URW, where the Hit was found to interact with the important residues seated at its active site. Our results also demonstrate that binding with Cys919 and Lue83 are important in designing a dual inhibitor for angiogenesis and progression. Taken together, we suggest that the Hit compound might be a potential lead candidate that can repress cancer angiogenesis and growth.

## Additional files

Additional file 1: Test set assessment. Assessing the test set values for estimated and the experimental activities by Hypo1. (DOCX 18 kb)

Additional file 2: Cocrystal re-dock results of 4AG8. Overlapping of the co-crystal (cyan) onto the docked pose (orange). The binding pattern was found to be similar. (DOCX 181 kb)

Additional file 3: 2D interaction representation of the the reference compound and 4AG8. Detailed molecular interactions of the reference compound. (DOCX 420 kb)

Additional file 4: 2D interaction representation of the Hit compound and 4AG8. Detailed molecular interactions of the Hit compound. (DOCX 424 kb)

Additional file 5: Docking of the co-crystal within the binding pocket of 1URW. Docking of the co-crystal within the binding pocket. Pink is the docked pose and green represents the co-crystal position. (DOCX 142 kb)

Additional file 6: 2D interaction representation of the co-crystal and 1URW. Detailed molecular interactions of the co-crystal compound. (DOCX 229 kb)

Additional file 7: 2D interaction representation of the reference compound and 1URW. Molecular interaction details of the reference compound. (DOCX 204 kb)

Additional file 8: 2D interaction representation of the Hit compound and 1URW. Molecular interaction details of the Hit compound. (DOCX 264 kb)

Additional file 9: Active sites comparison. Comparison of the active site residues of 4AG8 and 1UMR. (DOCX 13 kb)

## Abbreviations

2D: Two Dimensiional
3D QSAR: Three-dimensional quantitative structure-activity relationships
3D: Three Dimensional
Å: Angstrom
A: Number of actives
ACD: Advanced chemistry development
ADMET: Absorption, distribution, metabolism, excretion, toxicity
ATP: Adenosine Triphosphate
B3LYP: Becke, 3-parameter, Lee-Yang-Parr
BBB: Blood Brain Barrier
CDK: Cyclin-dependent kinases
CHARMm: Chemistry at HARvard Macromolecular Mechanics
CYPs: Cytochromes P450
D: Total number of molecules in a database
DFT: Density functional theory
DGF: Asp-Phe-Gly
DS: Discovery Studio
EF: Enrichment Factor
Ff: force field
G1: Gap 1
GF: Goodness of Fit
GOLD: Genetic optimisation for ligand docking
GROMACS: GROningen machine for chemical simulations
Ha: Total Actives
HBA: Hydrogen bond acceptor
HBD: Hydrogen bond donor
HIA: Human intestinal absorption
HOMO: Highest occupied Molecular Orbitals
Ht: Total Hits
Hy Ali: Hydrophobic aliphatic
HyP: HydroPhobic
IC_50_: Half maximal inhibitory concentration
LINCS: Linear constraint solver for molecular simulations
LUMO: Lowest unoccupied molecular orbitals
Max: Maximum
MD: Molecular dynamics
NCI: National cancer institute
NPT: Number of particles, pressure, and temperature
NVT: Number of particles, volume, and temperature
PDB: Protein data bank
PM: Pocket match
PME: Patricle mesh ewald
PPB: Plasma protein binding
RA: Ring aromatic
RKT: Receptor Tyrosine Kinases
RMSD: Root mean square deviation
S phase: Synthesis phase
TIP3P: Three-site transferrable intermolecular potential
VEGFRs: Vascular endothelial growth factor receptors
VEGFs: Vascular endothelial growth factors
VMD: Visual molecular dynamics
