# Correction to: Exploration for novel inhibitors showing back-to-front approach against VEGFR-2 kinase domain (4AG8) employing molecular docking mechanism and molecular dynamics simulations

**DOI:** 10.1186/s12885-019-6378-6

**Published:** 2019-12-26

**Authors:** Shailima Rampogu, Ayoung Baek, Amir Zeb, Keun Woo Lee

**Affiliations:** Division of Applied Life Science (BK21 Plus Program), Systems and Synthetic Agrobiotech Center (SSAC), Plant Molecular Biology and Biotechnology Research Center (PMBBRC), Research Institute of Natural Science (RINS), Gyeongsang National University (GNU), 501 Jinju-daero, Jinju, 52828 Republic of Korea

**Correction to: BMC Cancer (2018) 18:264**

**https://doi.org/10.1186/s12885-018-4050-1**

Following publication of the original article [1], the authors reported errors in Fig. 3, Fig. 14a, Fig. 18, Fig. 19b, Additional file 3 and Additional file 7. The title of Additional file 9 contains a typing error and is correctly given below.

**Fig. 3:**
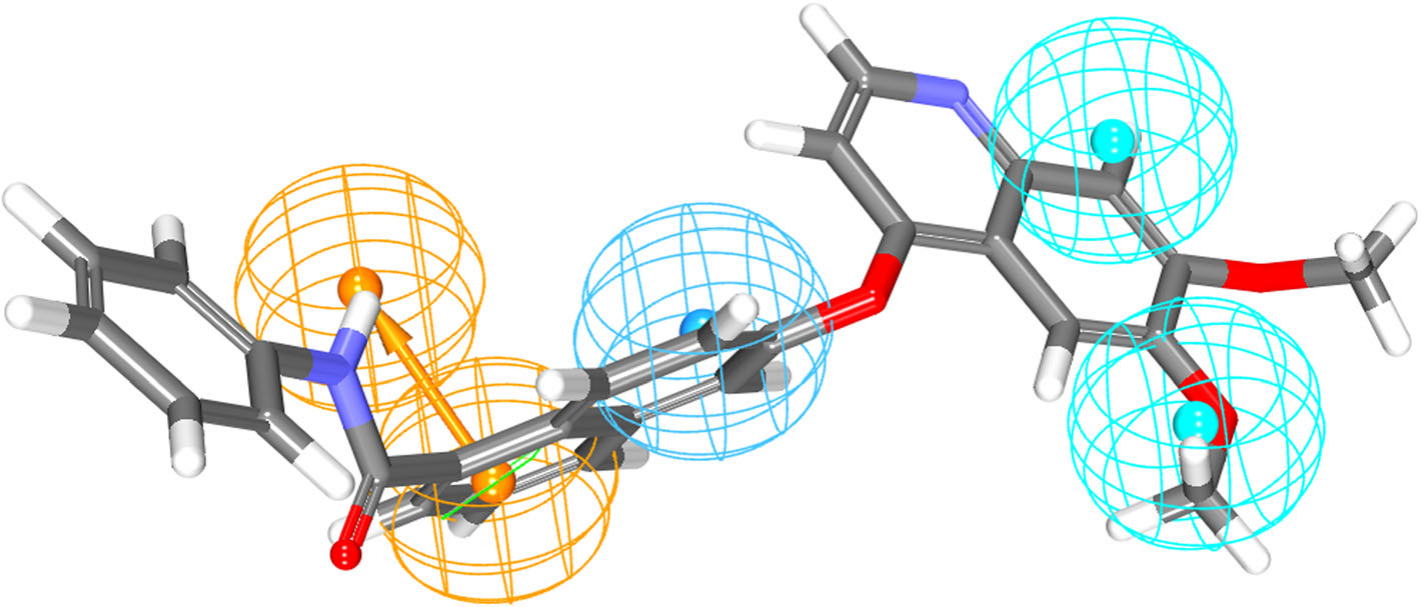
Most active compound (IC_50_ = 0.2) mapped to all the features

**Fig. 14:**
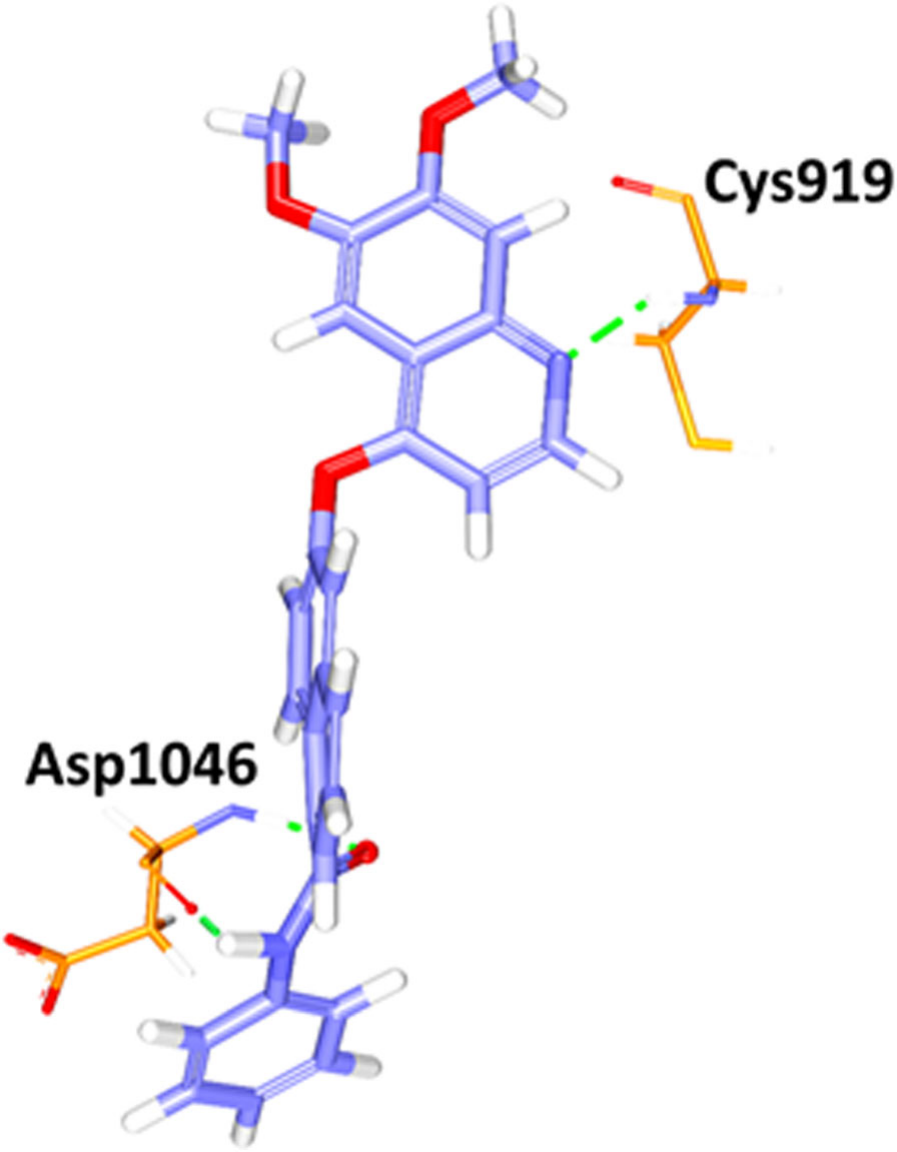
a Molecular interaction between the reference- protein (purple). Green dotted lines indicate the hydrogen bonds. The residues are represented in orange stick model

**Fig. 18:**
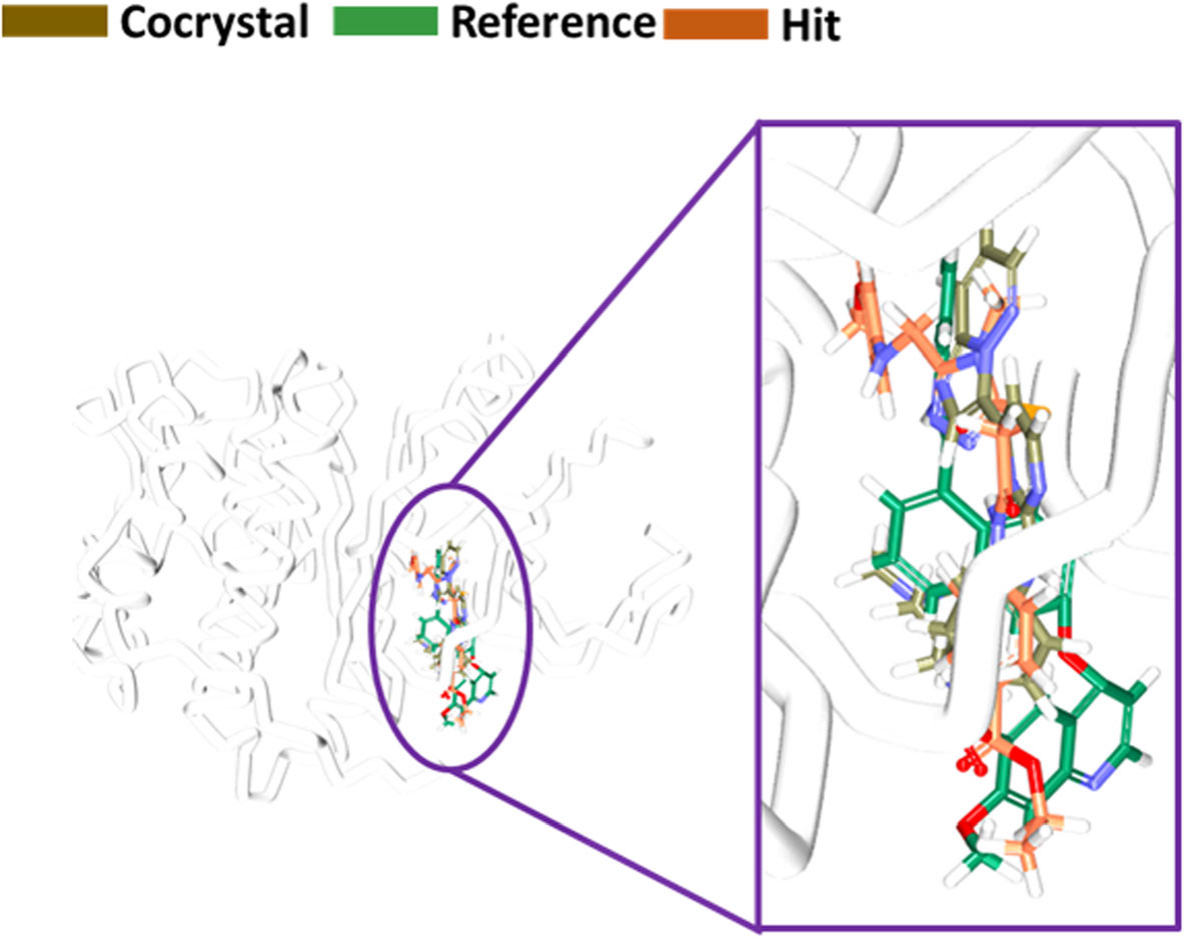
Binding mode assessment of compounds. The co-crystal is represented in gray, reference is denoted in green and the Hit in orange. All the three follow the same pattern

**Fig. 19:**
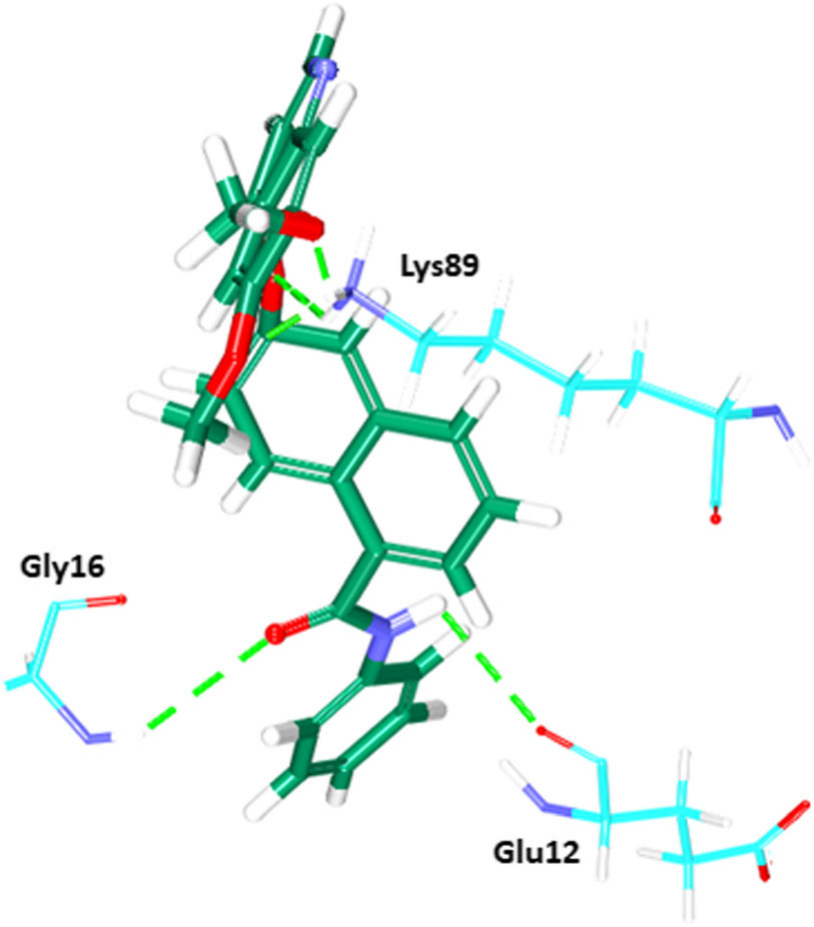
b Intermolecular interactions between the ligand and the protein. Green dotted lines represent the hydrogen bonds. The protein residues are indicated in cyan

The following typing errors have been identified:

**Table:** 

Page no	Column/paragraph	line	Present word	Change to
1	Abstract/results	2	of	above
2	1/2	2	Cyclic	Cyclin
2	2/1	3	VEGFR	VEGFR-2
12	2/1	3	prognosis	progression
12	2/1	10	form	from
19	Above conclusions	4	Cyc919	Cys919
10	2/1	13	20 ps	20 ns
10	2/1	15	25 ps	25 ns
13	2/1	6	four	five
Table 5/ reference	van der Waals interactions	–	asn900leu1044	Asn900, Ile1044
Fig 10	–	–	30 ps	30 ns
Fig 16	–	–	refrence	reference

Further to this, in Table 1, HyP is incorrectly represented as HyB and Hy-Ali as HyAli/HY-Ali. The corrected Table 1 can be found here.

**Table 1:** Table 1

Hypo no	Total cost^a^	Cost difference	RMSD^b^	Correlation	Features^c^	Max fit
Hypo1	111.95	71.22	0.7	0.97	Hy-Ali, 2HyP,RA	11.4
Hypo 2	113.31	69.86	0.7	0.96	Hy-Ali, 2HyP,RA	11.5
Hypo 3	116.45	66.71	0.8	0.95	Hy-Ali,HyP,RA,HBA	11.9
Hypo 4	116.47	66.69	1.0	0.94	HBA, HBD 2HyP	10.7
Hypo 5	117.11	66.05	0.9	0.94	Hy-Ali,HyP,RA,HBA	11.5
Hypo 6	119.51	63.65	1.0	0.93	HBA,HBD,2HyP	11.26
Hypo 7	119.52	63.65	0.9	0.95	HBA,2HyP,RA	12.65
Hypo 8	119.82	63.35	0.9	0.94	HBA,Hy-Ali,HBD,RA	12.33
Hypo 9	119.94	63.23	1.2	0.91	HBA,Hy-Ali,2HyP, RA	11.98
Hypo10	120.52	62.65	1.1	0.91	HBA,HBD, Hy-Ali	7.8

These corrections do not alter the context of the manuscript.

## Supplementary information

**Additional file 3.** 2D interaction representation of the reference compound and 4AG8. Detailed molecular interactions of the reference compound.

**Additional file 7.** 2D interaction representation of the reference compound and 1URW. Molecular interaction details of the reference compound.

**Additional file 9.** Active sites comparison. Comparison of the active site residues of 4AG8 and 1URW.
