# Supplementary material for: Exploration for novel inhibitors showing back-to-front approach against VEGFR-2 kinase domain (4AG8) employing molecular docking mechanism and molecular dynamics simulations
Source: BMC Cancer. 2018 Mar 7;18:264. doi: 10.1186/s12885-018-4050-1 (PMC5842552; doi:10.1186/s12885-018-4050-1)
Supplement: Supplementary file 1 — Test set assessment. Assessing the test set values for estimated and the experimental activities by Hypo1. (DOCX 18 kb) [file 12885_2018_4050_MOESM1_ESM.docx]

| Name | Fit value | Experimental IC50 (nmol/L) | Predicted IC50 (nmol/L) | Error^a^ | Experimental Scale | Predicted Scale |
| --- | --- | --- | --- | --- | --- | --- |
| Compound1 | 6.89565 | 0.2 | 0.29 | 1.49 | +++ | +++ |
| Compound2 | 6.07665 | 0.8 | 1.97 | 2.46 | +++ | +++ |
| Compound3 | 5.84561 | 3 | 3.36 | 1.12 | +++ | +++ |
| Compound4 | 5.80688 | 4 | 3.67 | -1.08 | +++ | +++ |
| Compound5 | 5.34265 | 16 | 10.69 | -1.49 | +++ | +++ |
| Compound6 | 4.68257 | 27 | 48.91 | 1.81 | +++ | +++ |
| Compound7 | 5.41171 | 47 | 9.12 | -5.15 | +++ | +++ |
| Compound8 | 4.51219 | 50 | 72.41 | 1.44 | +++ | +++ |
| Compound9 | 4.66187 | 51 | 51.3 | 1.00 | +++ | +++ |
| Compound10 | 5.1096 | 76 | 18.29 | -4.15 | +++ | +++ |
| Compound11 | 4.63195 | 79 | 54.96 | -1.43 | +++ | +++ |
| Compound12 | 4.56058 | 79 | 64.77 | -1.21 | +++ | +++ |
| Compound13 | 4.61412 | 83 | 57.2 | -1.44 | +++ | +++ |
| Compound14 | 4.66527 | 84 | 50.9 | -1.65 | +++ | +++ |
| Compound15 | 4.63649 | 92 | 54.38 | -1.69 | +++ | +++ |
| Compound16 | 4.5794 | 100 | 62.03 | -1.61 | +++ | +++ |
| Compound17 | 4.64843 | 102 | 52.91 | -1.92 | +++ | +++ |
| Compound18 | 4.67559 | 114 | 49.7 | -2.29 | +++ | +++ |
| Compound19 | 4.63701 | 120 | 54.32 | -2.2 | +++ | +++ |
| Compound20 | 4.55141 | 120 | 66.15 | -1.81 | +++ | +++ |
| Compound21 | 4.59647 | 123 | 59.63 | -2.06 | +++ | +++ |
| Compound22 | 4.4013 | 125 | 93.47 | -1.33 | +++ | +++ |
| Compound23 | 4.52378 | 128 | 70.5 | -1.81 | +++ | +++ |
| Compound24 | 4.48615 | 130 | 76.88 | -1.690 | +++ | +++ |
| Compound25 | 4.49085 | 136 | 76.05 | -1.78 | +++ | +++ |
| Compound26 | 5.1226 | 140 | 17.75 | -7.88 | +++ | +++ |
| Compound27 | 4.62277 | 140 | 56.13 | -2.49 | +++ | +++ |
| Compound28 | 4.52706 | 140 | 69.97 | -2.00 | +++ | +++ |
| Compound29 | 4.4767 | 145 | 78.57 | -1.8 | +++ | +++ |
| Compound30 | 4.68205 | 215 | 48.97 | -4.39 | +++ | +++ |
| Compound31 | 4.6969 | 230 | 47.32 | -4.85 | +++ | +++ |
| Compound32 | 4.66829 | 231 | 50.54 | -4.56 | +++ | +++ |
| Compound33 | 4.65253 | 268 | 52.4162 | -5.112 | ++ | +++ |
| Compound34 | 4.57551 | 280 | 62.58 | -4.47374 | ++ | +++ |
| Compound35 | 4.66169 | 455 | 51.32 | -8.86539 | ++ | +++ |
| Compound36 | 3.39513 | 2,089 | 948.12 | -2.20329 | ++ | ++ |
| Compound37 | 3.52573 | 4,074 | 701.8 | -5.80437 | ++ | ++ |
| Compound38 | 2.35742 | 1,000,000 | 10,341.40 | -96.6987 | + | + |
| Compound39 | 2.34909 | 1,000,000 | 10,541.60 | -94.8623 | + | + |

^a^Error, ratio of the predicted activity (Pred IC50) to the experimental activity (Exp IC50) or its negative inverse if the ratio is <1.
