# Supplementary figures and images for: Exploration for novel inhibitors showing back-to-front approach against VEGFR-2 kinase domain (4AG8) employing molecular docking mechanism and molecular dynamics simulations
Source: BMC Cancer. 2018 Mar 7;18:264. doi: 10.1186/s12885-018-4050-1 (PMC5842552; doi:10.1186/s12885-018-4050-1)

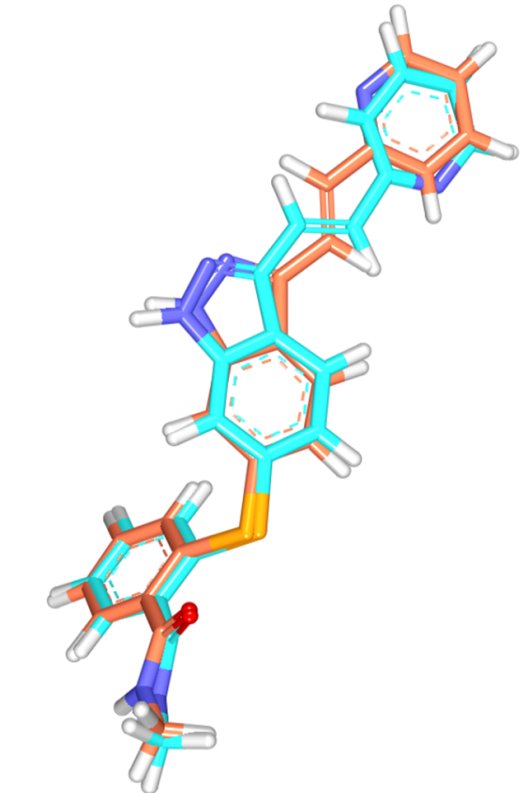

Supplement: Supplementary file 2 — Cocrystal re-dock results of 4AG8. Overlapping of the co-crystal (cyan) onto the docked pose (orange). The binding pattern was found to be similar. (DOCX 181 kb) [file 12885_2018_4050_MOESM2_ESM.docx]

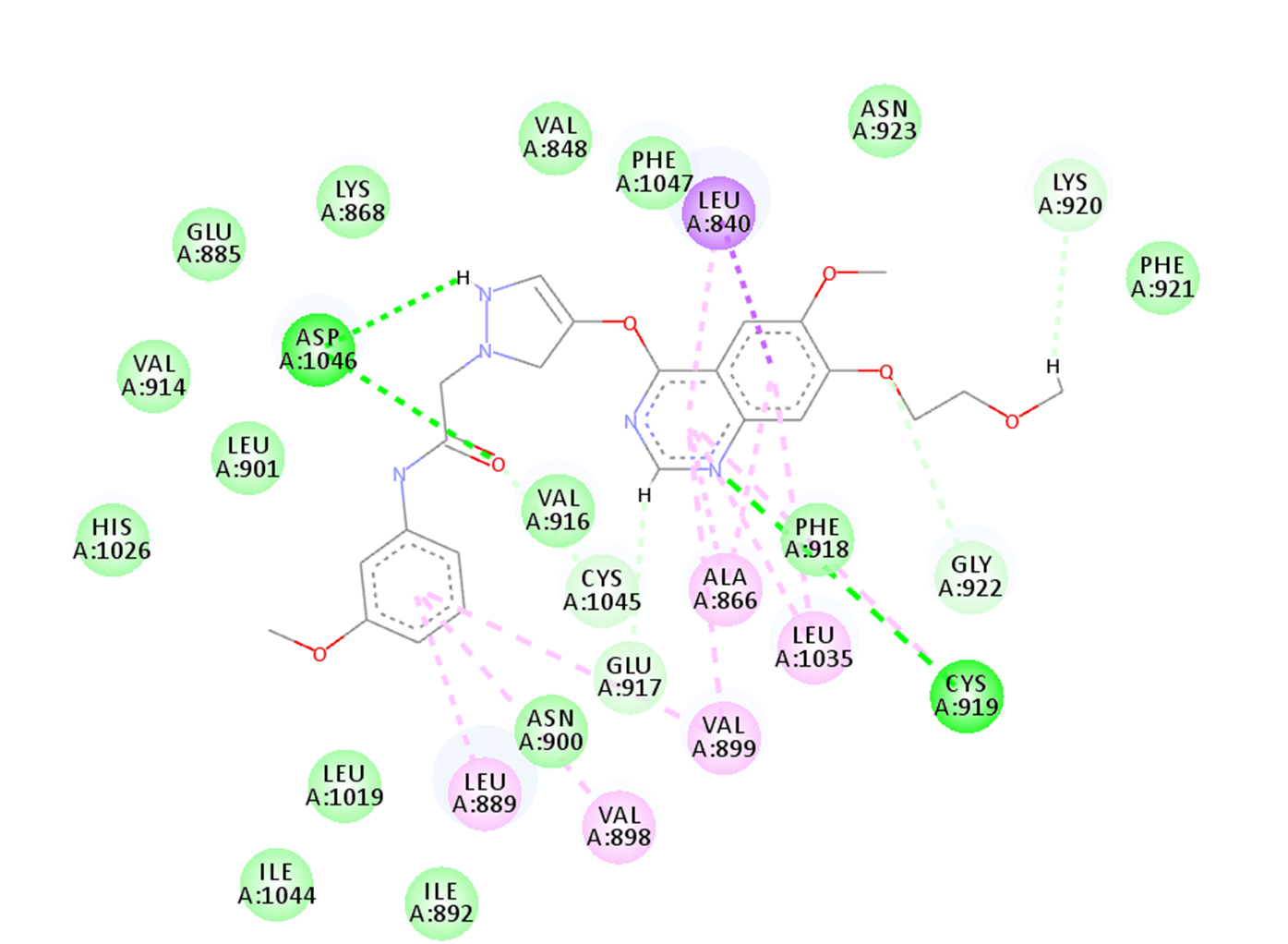

Supplement: Supplementary file 3 — 2D interaction representation of the the reference compound and 4AG8. Detailed molecular interactions of the reference compound. (DOCX 420 kb) [file 12885_2018_4050_MOESM3_ESM.docx]

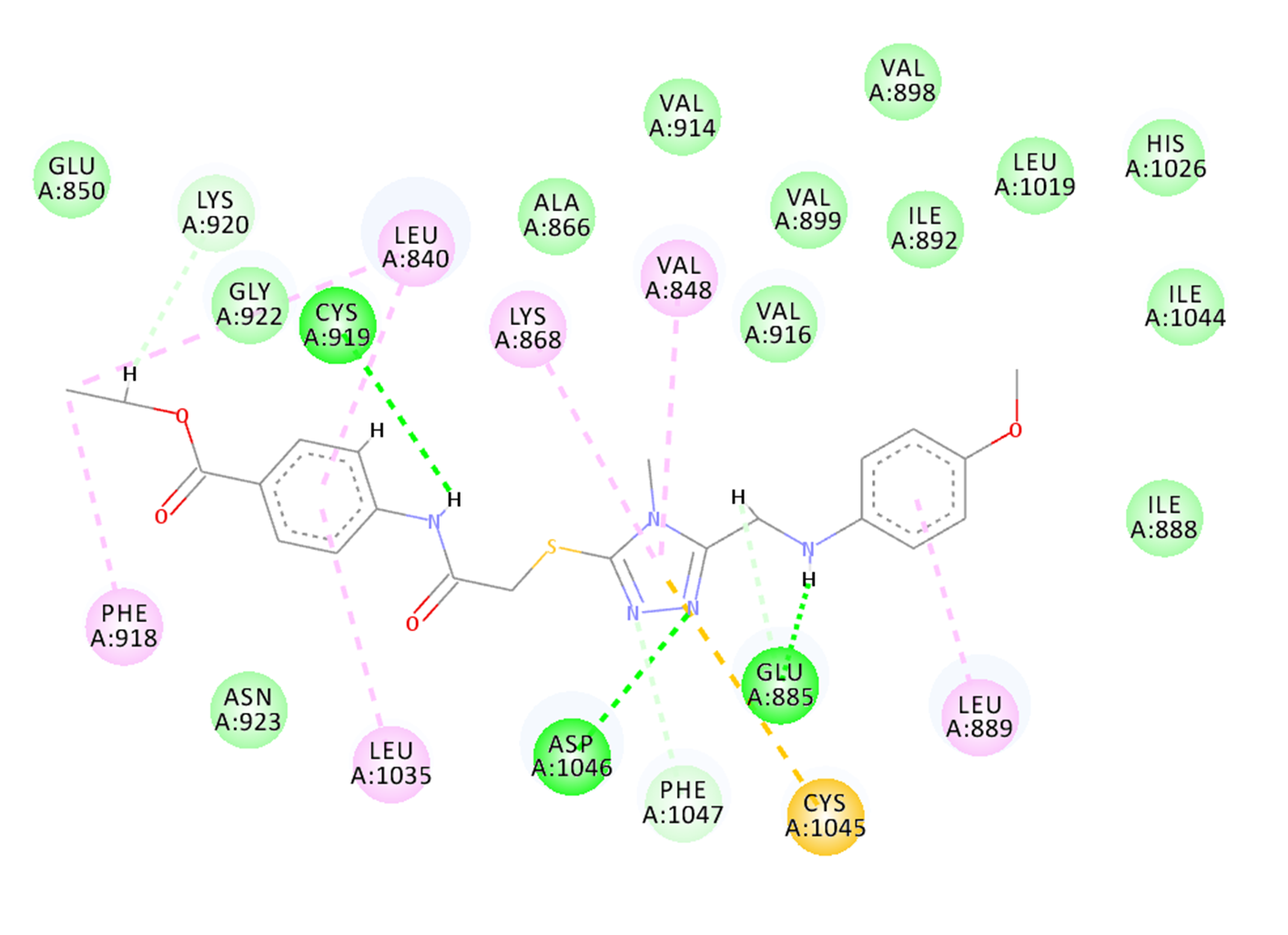

Supplement: Supplementary file 4 — 2D interaction representation of the Hit compound and 4AG8. Detailed molecular interactions of the Hit compound. (DOCX 424 kb) [file 12885_2018_4050_MOESM4_ESM.docx]

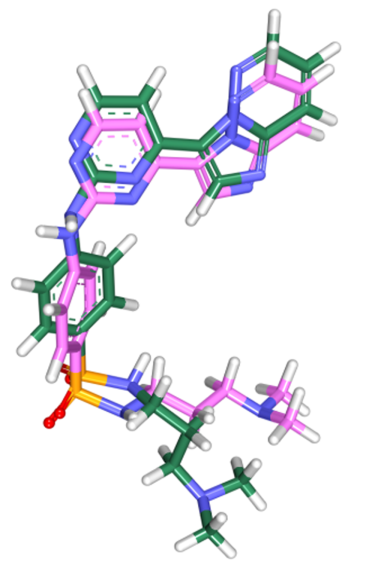

Supplement: Supplementary file 5 — Docking of the co-crystal within the binding pocket of 1URW. Docking of the co-crystal within the binding pocket. Pink is the docked pose and green represents the co-crystal position. (DOCX 142 kb) [file 12885_2018_4050_MOESM5_ESM.docx]

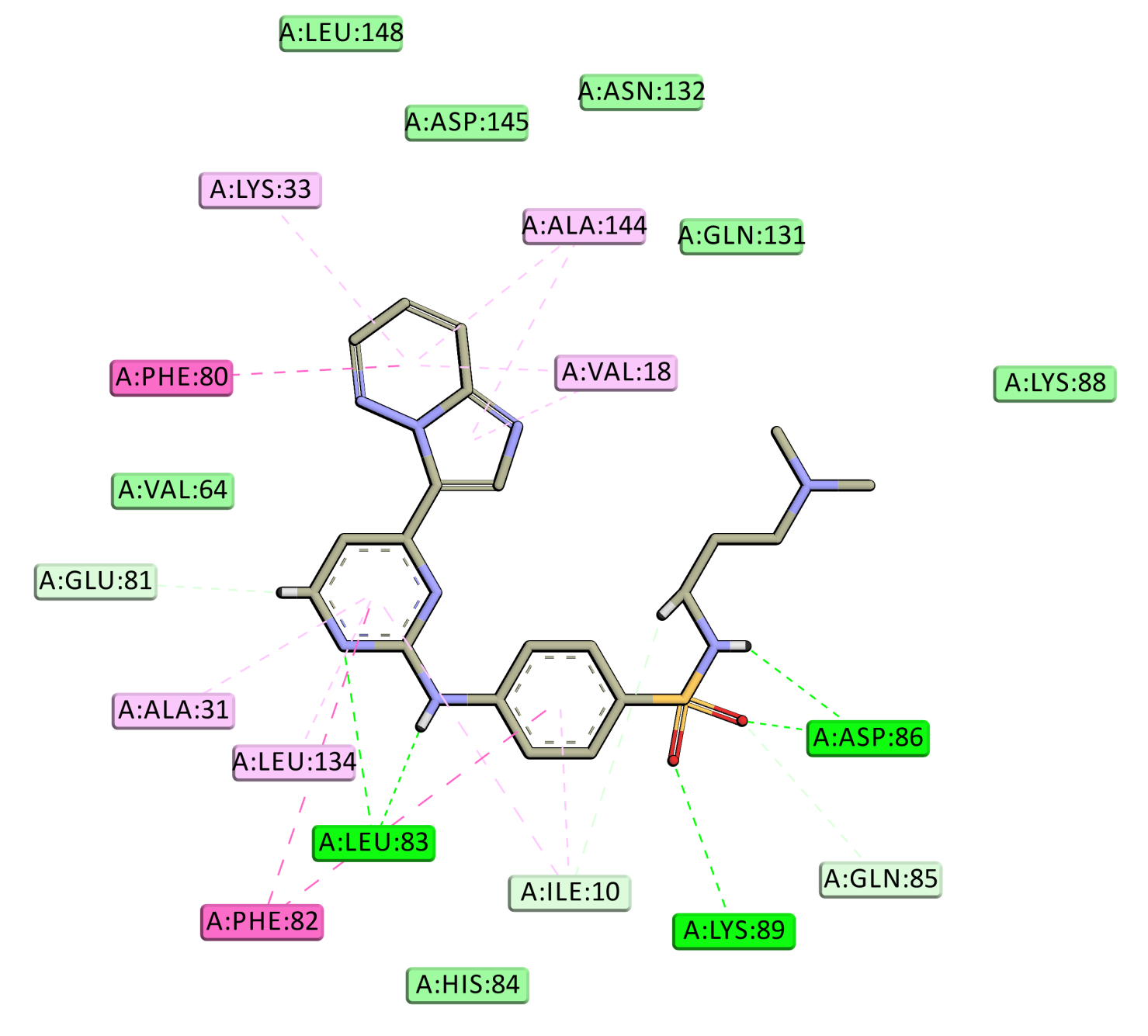

Supplement: Supplementary file 6 — 2D interaction representation of the co-crystal and 1URW. Detailed molecular interactions of the co-crystal compound. (DOCX 229 kb) [file 12885_2018_4050_MOESM6_ESM.docx]

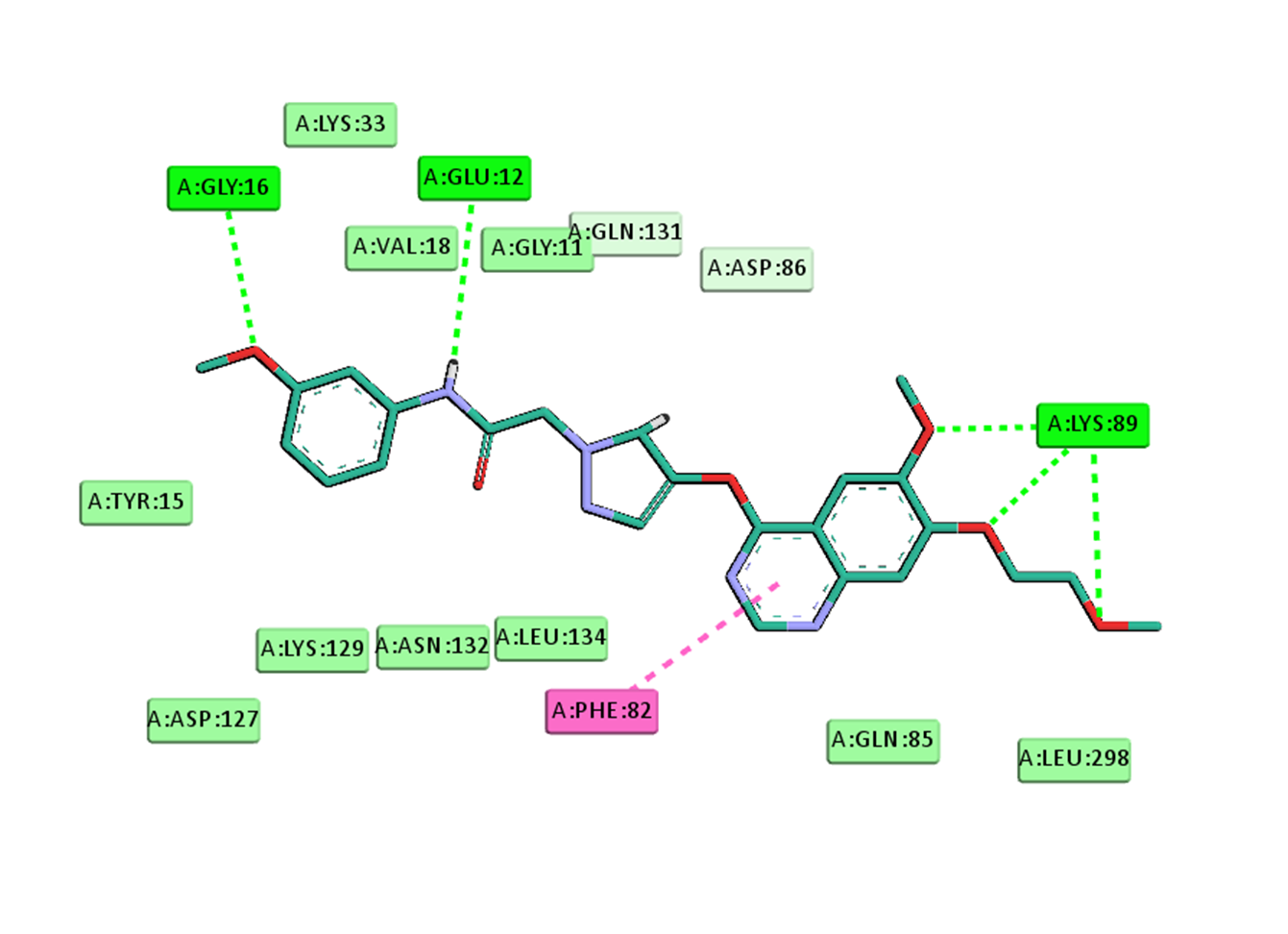

Supplement: Supplementary file 7 — 2D interaction representation of the reference compound and 1URW. Molecular interaction details of the reference compound. (DOCX 204 kb) [file 12885_2018_4050_MOESM7_ESM.docx]

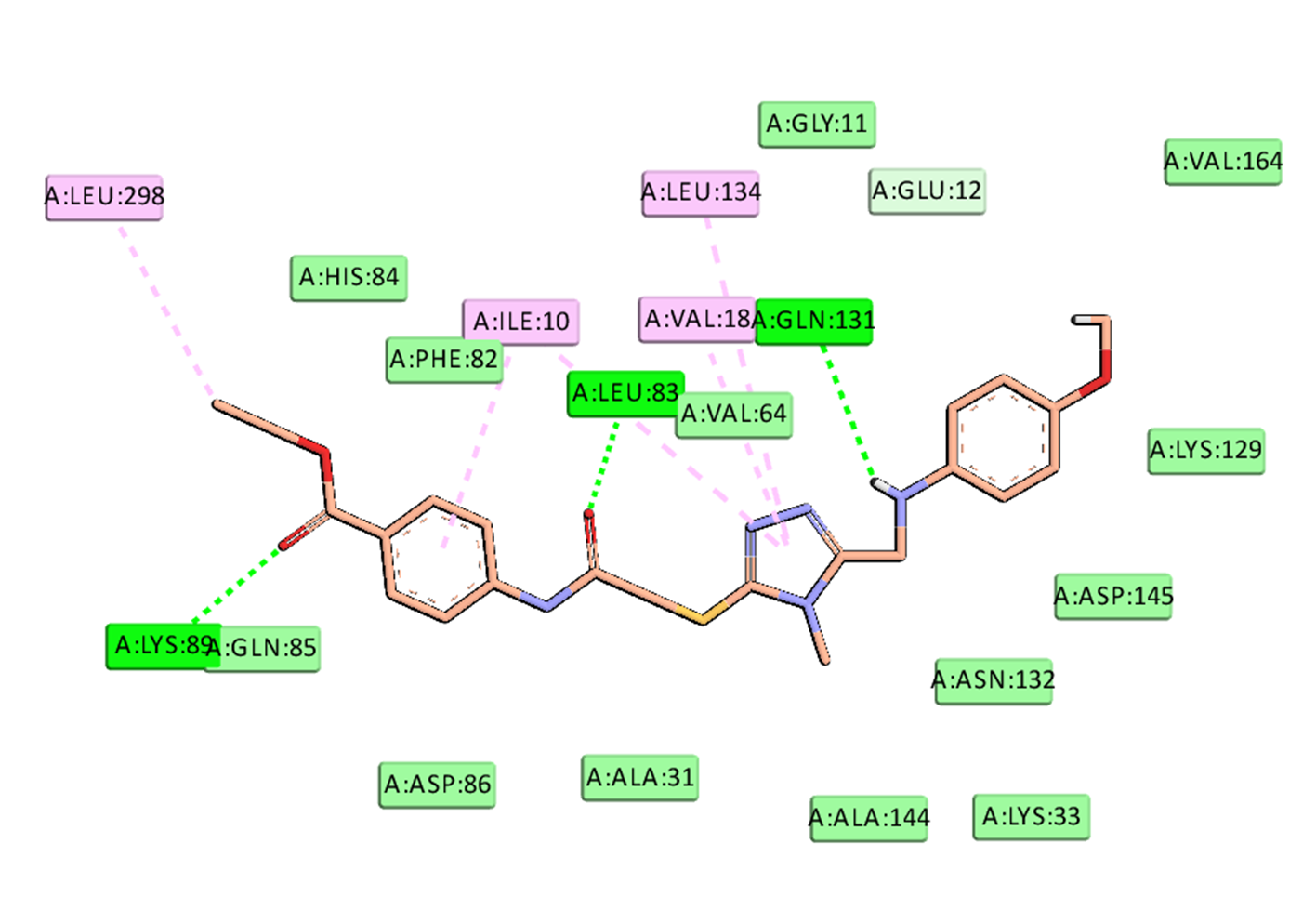

Supplement: Supplementary file 8 — 2D interaction representation of the Hit compound and 1URW. Molecular interaction details of the Hit compound. (DOCX 264 kb) [file 12885_2018_4050_MOESM8_ESM.docx]
