# Supplementary material for: Exploration for novel inhibitors showing back-to-front approach against VEGFR-2 kinase domain (4AG8) employing molecular docking mechanism and molecular dynamics simulations
Source: BMC Cancer. 2018 Mar 7;18:264. doi: 10.1186/s12885-018-4050-1 (PMC5842552; doi:10.1186/s12885-018-4050-1)
Supplement: Supplementary file 9 — Active sites comparison. Comparison of the active site residues of 4AG8 and 1UMR. (DOCX 13 kb) [file 12885_2018_4050_MOESM9_ESM.docx]

| 4AG8 | 1URW |
| --- | --- |
| Leu840 | Ile10 |
| Ala866 | Ala31 |
| Lys868 | Lys33 |
| Val898 | Val64 |
| Glu917 | Glu81 |
| Phe918 | Phe82 |
| Cys919 | Leu83 |
| Leu1035 | Leu134 |
| Asp1046 | Asp145 |
