# Supplementary material for: Correction to: Exploration for novel inhibitors showing back-to-front approach against VEGFR-2 kinase domain (4AG8) employing molecular docking mechanism and molecular dynamics simulations
Source: BMC Cancer. 2019 Dec 26;19:1249. doi: 10.1186/s12885-019-6378-6 (PMC6933660; doi:10.1186/s12885-019-6378-6)

2D interaction representation of the reference compound and 4AG8. Detailed molecular interactions of the reference compound.


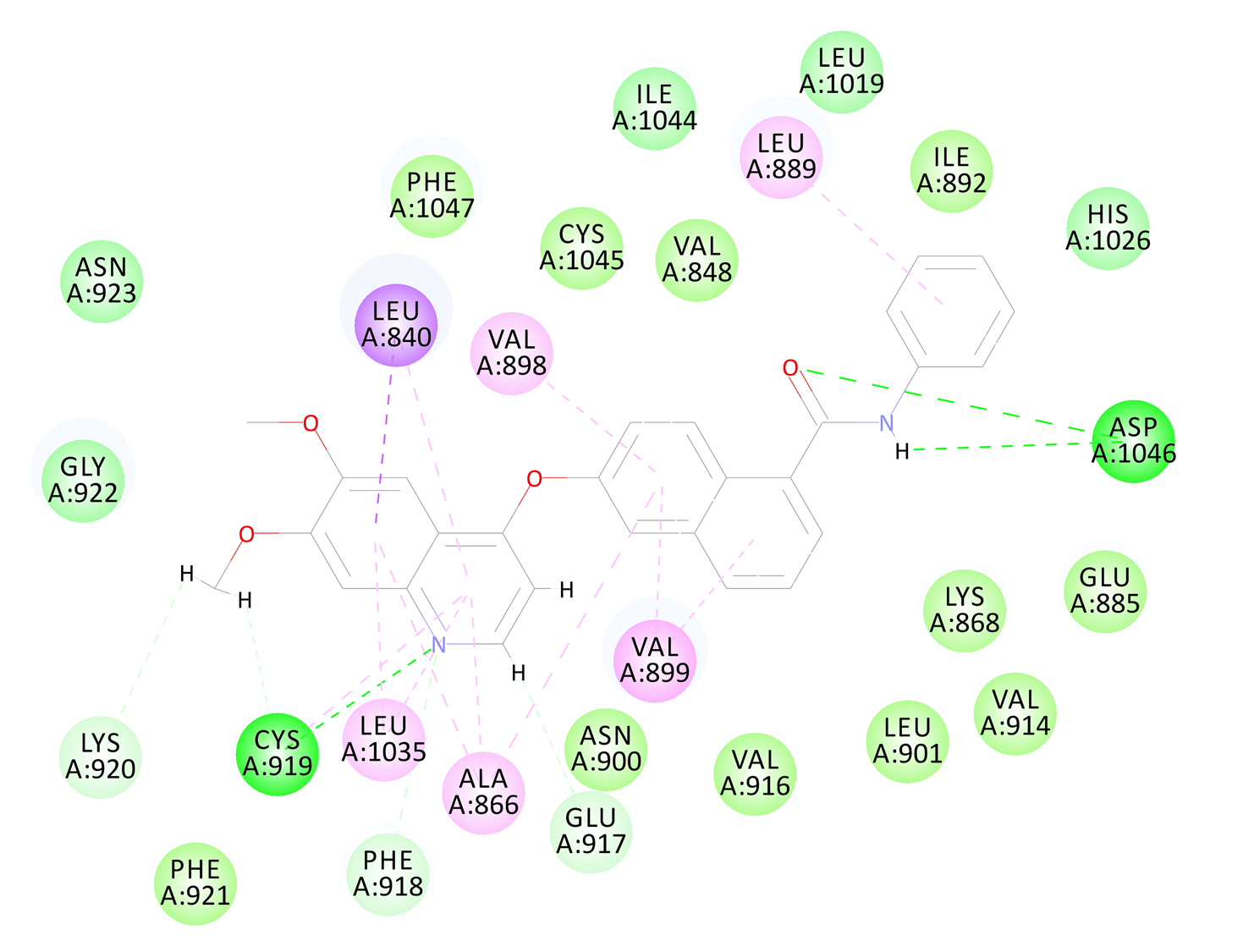

Supplement: Supplementary file 1 — Additional file 3. 2D interaction representation of the reference compound and 4AG8. Detailed molecular interactions of the reference compound. [file 12885_2019_6378_MOESM1_ESM.docx]
