# Supplementary material for: Correction to: Exploration for novel inhibitors showing back-to-front approach against VEGFR-2 kinase domain (4AG8) employing molecular docking mechanism and molecular dynamics simulations
Source: BMC Cancer. 2019 Dec 26;19:1249. doi: 10.1186/s12885-019-6378-6 (PMC6933660; doi:10.1186/s12885-019-6378-6)
Supplement: Supplementary file 3 — Additional file 9. Active sites comparison. Comparison of the active site residues of 4AG8 and 1URW. [file 12885_2019_6378_MOESM3_ESM.docx]

Active sites comparison. Comparison of the active site residues of 4AG8 and 1URW

| 4AG8 | 1URW |
| --- | --- |
| Leu840 | Ile10 |
| Ala866 | Ala31 |
| Lys868 | Lys33 |
| Val898 | Val64 |
| Glu917 | Glu81 |
| Phe918 | Phe82 |
| Cys919 | Leu83 |
| Leu1035 | Leu134 |
| Asp1046 | Asp145 |
